# QTL Mapping for Phosphorus Efficiency and Morphological Traits at Seedling and Maturity Stages in Wheat

**DOI:** 10.3389/fpls.2017.00614

**Published:** 2017-04-24

**Authors:** Yuanyuan Yuan, Minggang Gao, Mingxia Zhang, Honghua Zheng, Xiuwen Zhou, Ying Guo, Yan Zhao, Fangmei Kong, Sishen Li

**Affiliations:** ^1^State Key Laboratory of Crop Biology, National Engineering Laboratory for Efficient Utilization of Soil and Fertilizer Resources, Shandong Agricultural UniversityTai'an, China; ^2^Jinan Academy of Agricultural ScienceJinan, China; ^3^Key Laboratory of Biochemistry and Molecular Biology, College of Biological and Agricultural Engineering, Weifang UniversityWeifang, China

**Keywords:** common wheat, phosphorus efficiency (PE), morphological trait, quantitative trait locus (QTL), recombinant inbred line (RIL), EST sequences

## Abstract

Phosphorus (P) efficiency (PE), which comprises phosphorus uptake (PupE) and utilization efficiency (PutE), is considered as one of the most important factors for crop yield. In the present study, 11 seedling traits and 13 maturity traits related to wheat PE and morphology were investigated using a set of recombinant inbred lines (RILs) derived from the cross of “TN 18 × LM 6,” under hydroponic culture trials and field trials at low P (LP) and normal P (NP) levels in two different years, respectively. The LP input reduced of biomass, yield and PupE traits, but increased PutE traits. A total of 163 QTLs for seedling and maturity traits under different P levels and their AV, and 15 QTLs for relative traits were detected on 21 chromosomes. Of these, 49 and 63 QTLs for were detected specially in LP and NP treatments, respectively. We found 11 relatively high-frequency QTLs (RHF-QTLs) and four important QTL clusters, which may be the potential targets for marker-assisted selection (MAS) in wheat breeding programs for PE. Favorable relationships for breeding programs were found in the four important QTL clusters, which allow the possibility of improving the morphological traits and PutE simultaneously. A total of 29 markers which associated with 51 QTLs were found highly homologous with EST sequences, which suggested that they were potential functional loci. We suggested that the four biomass traits (SDW, RDW, TDW, and RSDW), five yield traits (SN, PH, TGW, GWP, and StWP) and two relative traits (Rstwp and Rgwp) can be considered as the primary indexes for the evaluation of PE for they are easy to identify on a large-scale.

## Introduction

As one of the most important staple crops worldwide, common wheat (*Triticum aestivum* L.) grows on over 216 million hectares and produces over 675 million metric tons (http://faostat.fao.org). The productivity and quality of wheat is very important for agricultural sustainable development and the food supply. Phosphorus (P) is one of the three most important nutrients for the growth and yield improvement of wheat (Ozturk and Cakmak, 2005; Malhi et al., 2015). However, the winter wheat-producing areas in China are mostly distributed in calcareous soils, and an insufficient supply of available P in these soils is one of the main limiting factors of wheat production. Approximately 103 kg ha^−1^ of P fertilizers (P_2_O_5_) is supplied every year for wheat productivity in China (Su et al., 2009). Heavy P fertilizers applications to soils has increased crop production costs, exhausted non-renewable P resources, and caused a series of environmental problems (Peleg et al., 2009; Bayuelo-Jiménez et al., 2011; Dawson and Hilton, 2011; Kai et al., 2014). At the same time, P fertilizer use efficiency is merely approximately 10% in wheat and most of the applied P is fixed in the soil (Schröder et al., 2011). Fortunately, P efficiency (PE) has shown a significant genotypic differences and has been widely reported in wheat (Batten, 1994; Ozturk and Cakmak, 2005; Liao et al., 2008; Malhi et al., 2015; Nisar et al., 2016). Developing wheat cultivars with high PE is a desirable solution to reduce P fertilizer and make full use of soil P, and may offer a sustainable solution to manage P nutrition in wheat production (Baker et al., 2015; Vandamme et al., 2016). However, the genetic basis of P uptake and utilization efficiency is still poorly understood.

PE has been described as the proportion of yield potential that can be achieved under P deficiency stress, which has two components of P uptake efficiency (PupE) and P utilization efficiency (PutE) (Siddiqi and Glass, 1981; Meng et al., 2014; McDonald et al., 2015). High PE genotypes usually have a high capacity to take up relatively more P in P-deficient soil (PupE) and/or high ratio of biomass and tissue P nutrient concentration (PutE) (Guo et al., 2012; Kong et al., 2013). P efficiency-related traits are typically quantitative traits (James et al., 2016; van de Wiel et al., 2016). In recent decades, a number of quantitative trait loci (QTLs) for PE and related traits have been identified and mapped on all 21 chromosomes of wheat under hydroponic culture trials (Guo et al., 2012; Zhang and Wang, 2015), pot trials (Su et al., 2006; Ryan et al., 2015) and field trials (Su et al., 2009), and some relatively high frequency QTLs (RHF-QTLs) and important QTL clusters in the same genomic regions were detected. For example, Su et al. (2006) found three QTL clusters for four P-related traits on chromosomes 4B, 5A, and 5D; they also detected seven and six QTLs repeatedly as controlling P uptake and utilization efficiency, respectively (Su et al., 2009). Guo et al. (2012) identified 32 RHF-QTLs, which were expressed in 4–10 different N, P, and K treatments, and mapped 26 important QTL clusters on 13 chromosomes: 1A, 1B, 1D, 2B, 3A, 3B, 4A, 4B, 5D, 6A, 6B, 7A, and 7B. Zhang and Wang (2015) using three recombinant inbred line (RIL) populations with a common female parent detected 28 major QTLs in multiple populations or under different P treatments, and 18 important QTL clusters were mapped on 12 chromosomes: 1D, 2A, 2B, 3A, 3B, 4B, 4D, 5A, 5D, 6A, 6B, and 7B. Ryan et al. (2015) using the RIL and doubled haploid line (DH) populations identified seven and nine QTLs for shoot biomass respectively, and three major QTLs were distributed on chromosomes 4A, 4B, and 7A.

QTL analysis was conducted mainly using RIL or DH populations derived from the cross of two parents (Su et al., 2006, 2009; Guo et al., 2012; Ryan et al., 2015; Zhang and Wang, 2015). Considering the utilization of QTLs in breeding programs, it is favorable to select a cultivated variety or a core parent as one parents of the RIL or DH population. We constructed a set of RILs derived from a cross of “Tainong 18 × Linmai 6” (TN18 × LM6, TL-RILs), and obtained a high-density genetic map (Zhang, 2014). TN18 is a cultivated variety and core parent developed by our group, and LM6 is an elite line.

In this study, the TL-RILs along with a high-density map were used to investigate the PE and morphological traits under different P levels in hydroponic culture and field trials across different years. The main objectives were to locate QTLs, and find the relatively stable QTLs and important QTL clusters that may be used in QTL cloning and wheat breeding programs.

## Materials and methods

### Plant materials

The RIL population used in the study was derived by single-seed descent (SSD) from a cross of “TN18 × LM6” (F_9_ in 2013). TN18 is a cultivated variety that was released in 2008 and is planted approximately 300 thousand hectare per year in the Huang-huai Winter Wheat Region, China. TN18 possesses several salient features, such as resistance to lodging, high grain yield and fine quality. TN18 is a semi-dwarf habit with about 75 cm in plant height, which is lower than most cultivated varieties. The *Rht* gene in TN18 is *Rht-B1b* came from the variety “Norin 10” by pedigree analysis. The male parent LM6 is an elite breeding line developed by the Linyi Academy of Agricultural Science, China. Two parents have distinct difference in PE: the PupE and PutE of LM6 is higher than TN18 at maturity stage in most instances. A total of 184 lines of the RILs that were randomly selected from the original 305 lines were used to conduct the study.

### Experimental design and trait measurement

#### Hydroponic culture trials at the seedling stage

Two independent hydroponic culture trials in two continuous years (2013 and 2014) were carried out under low and normal P (LP and NP, respectively) conditions with four replications for each treatment in a greenhouse (Table 1). Hoagland's nutrient solution (Hoagland and Arnon, 1950) was used with some amendment to achieve satisfactory growth for wheat (Table S1). The experiments adopted a randomized complete block design.

**Table 1:** **P treatments for the hydroponic culture and field trials**.

**Experimental design**	**Treatments**^**a**^					
	**Years**	**Trials**	**Names^a^**	**Codes**	**Times**	**P concentrations (H)/Replenishment (F)**
Hydroponic culture trial (H)	2013	1	NP	NP1	1 × P	0.2 mM
			LP	LP1	1/10 × P	0.02 mM
	2014	2	NP	NP2	1 × P	0.2 mM
			LP	LP2	1/10 × P	0.02 mM
Field trial (F)	2012–2013	3	NP	NP3		92 kg ha^−1^
			LP	LP3	Soil available P	0
	2013–2014	4	NP	NP4		102 kg ha^−1^
			LP	LP4	Soil available P	0

a *LP, low phosphorus; NP, normal phosphorus; NP1,NP2, NP3, and NP4, normal phosphorus treatment in trial 1, trial 2, trial 3 and trial 4 respectively; LP1, LP2, LP3, and LP4, low phosphorus treatment in trial 1, trial 2, trial 3, and trial 4 respectively*.

A total of 100 seeds for each line of the RILs and their parents were sterilized for 5 min in 10% H_2_O_2_, washed with distilled water, and germinated in Petri dishes with moist filter paper for 7 days. For each replication, two uniform seedlings for each line with both the embryogenic primary roots and coleoptiles (3–4 cm long), were selected. The seedlings were fixed with two sponges and transferred to a tray with holes placed on plastic tanks containing 20 L nutrient solution. The containers and tops for hydroponic culture were opaque to produce healthy roots and discourage algal growth. The distances between the different lines were 2 × 2 cm. The solution was continuously aerated through rubber tubes connected to an air compressor, and the nutrient solution was renewed every 4 days (Kong et al., 2013).

The first trial (Trial 1) was carried out from November 25 to December 31 in 2013. The temperature ranged from 8.1 to 30.7°C (average 16.0°C), relative humidity varied from 11.9 to 69.7% (average 49.4%), and a 9 h photoperiod was used (to obtain stronger seedlings) at 0.0–47.8 klux (average 4.1 klux). The second trial (Trial 2) was carried out from March 2 to April 6 in 2014. The temperature was between 8.0°C and 36.8°C (average 19.8°C), relative humidity was 5.0–82.9% (average 33.4%), and a 9 h photoperiod was employed at 0.0–50.4 klux (average 6.3 klux). Because of the huge quantity of work for the measurement of P concentration, all individual plants of each line for the four replications in the same P treatment were harvested together as one mixed sample and separated to two parts: root and shoot. All collected samples were oven-dried at 60°C for 72 h.

#### Field trials

We constructed eight 110 m^2^ (10 m × 11 m) nutrient plots at the Experimental Station of Shandong Agricultural University to perform the trials of the mineral nutrient elements. The plots were separated using a cement brick wall of 1.5 m in depth. The soil structure was maintained as nature field with a loamy soil, such that the soil conditions were the same as those in the field (Kong et al., 2013). The mineral nutrient elements were depleted by annually planting wheat and corn until the nutrient contents were in accordance with the demands of the trials.

Two field trials were conducted during the 2012-2013 (Trial 3) and 2013-2014 (Trial 4) growing seasons in the nutrient plots. The average N, P, and K in the 0–25 cm soil profile sampled before fertilization were 55.4, 23.3, and 84.4 mg kg^−^^1^ in 2012 and 63.6, 20.6, and 49.9 mg kg^−^^1^ in 2013, respectively. In both trials, two P treatments were used (Table 1). In Trial 3, the LP treatment was applied at 195 kg ha^−^^1^ N, 0 kg ha^−^^1^ P_2_O_5_ and 114 kg ha^−^^1^ K_2_O, and the NP treatment was applied at 195 kg ha^−^^1^ N, 92 kg ha^−^^1^ P_2_O_5_ and 114 kg ha^−^^1^ K_2_O. In the Trial 4, the LP treatment was applied at 182 kg ha^−^^1^ N, 0 kg ha^−^^1^ P_2_O_5_ and 198 kg ha^−^^1^ K_2_O, and the NP treatment was applied at 182 kg ha^−^^1^ N, 102 kg ha^−^^1^ P_2_O_5_ and 198 kg ha^−^^1^ K_2_O. All P_2_O_5_ and K_2_O and 60% of the N were applied before sowing, and 40% of the N was applied at the stem elongation stage. All 184 lines and their parents were grown in both LP and NP nutrient plots. Each trial was a complete block design with two replications in two nutrient plots, respectively. Each line was sown two rows with 1 m in long and 25 cm inter-row spacing. Twenty seeds for one row were sown with 5 cm spacing per plant. Seeds were sown on October 15, and plants were harvested on June 13–15.

#### Trait measurement

A summary of the trait measurement methods for all 30 investigated traits is presented in Table 2. For hydroponic culture trials, 11 traits were evaluated including four biomass traits (SDW, RDW, TDW, and RSDW), four PupE traits (SPC, RPC, TPC, and RSPC) and three PutE traits (SPutE, RPutE, and TPutE). The dry weight and P concentration for the root and shoot of each line were measured using the mixed sample of the same P treatment. For field trials, 13 traits were measured including nine yield traits (PH, SN, GN, SL, FSS, SSS, TGW, GWP, and StWP), two PupE traits (GPC and StPC), and two PutE traits (GPutE and StPutE). We harvested the aboveground parts of ten plants randomly for each line in each replication, and PH, SN, GN, SL, FSS, and SSS were determined from 10 random plants inside the row for each line in each replication; the other traits were measured using the mixed samples of the same P treatment after harvested.

**Table 2:** **Summary of investigated traits and their measurement methods under hydroponic culture and field trials**.

**Traits**		**Units**	**Methods of measurement**
**HYDROPONIC CULTURE TRIALS**			
SDW	Shoot dry weight per plant	mg·plant^−1^	Oven dried and weighted using 1/10,000
RDW	Root dry weight per plant		Balances
TDW	Total dry weight per plant	mg·plant^−1^	RDW + SDW
RSDW	Ratio of root and shoot dry weight	–	RDW/SDW
SPC	Shoot P-content per plant	mg·plant^−1^	Using a sequential plasma spectrometer
RPC	Root P-content per plant		(ICPS-7500, Japan).
TPC	Total P-content per plant	mg·plant^−1^	RPC + SPC
RSPC	Ratio of root and shoot content	–	RPC/SPC
SPutE	Shoot P-utilization efficiency	mg·(μg·mg^−1^)^−1^	SDW/[(SPC × 1,000)/SDW]
RPutE	Root P-utilization efficiency	mg·(μg·mg^−1^)^−1^	RDW/[(RPC × 1,000)/RDW]
TPutE	Total P-utilization efficiency	mg·(μg·mg^−1^)^−1^	TDW/[(TPC × 1,000)/TDW]
**FIELD TRAILS**			
PH	Plant height	cm	Average value of 10 random individual plants of each line in each replication
SN	Spike number per plant	–	
GN	Grain number per spike	–	
SL	Spike length	cm	
FSS	Fertile spikelet number per spike	–	
SSS	Sterile spikelet number per spike	–	
TGW	Thousand grain weight	g	Weighted three times of 200 grains for each line in each replication after harvested using 1/1,000 balances
GWP	Grain weight per plant	g·plant^−1^	Dried and weighted using 1/100 balances
StWP	Straw weight per plant	g·plant^−1^	Ditto
GPC	Grain P-content per plant	mg·plant^−1^	Using a sequential plasma spectrometer
StPC	Straw P-content per plant	mg·plant^−1^	(ICPS-7500, Japan)
GPutE	Grain P-utilization efficiency	g·(mg.g^−1^)^−1^	GWP/GPC/GWP
StPutE	Straw P-utilization efficiency	g·(mg.g^−1^)^−1^	StWP/StPC/StWP
**RELATIVE TRAITS**			
Rph	Relative trait for PH	–	PH under LP treatment vs. NP treatment
Rsn	Relative trait for SN	–	SN under LP treatment vs. NP treatment
Rgn	Relative trait for GN	–	GN under LP treatment vs. NP treatment
Rtgw	Relative trait for TWG	–	TWG under LP treatment vs. NP treatment
Rgwp	Relative trait for GWP	–	GWP under LP treatment vs. NP treatment
Rstwp	Relative trait for StWP	–	StWP under LP treatment vs. NP treatment

The relative traits were calculated by dividing the values of LP by the values of NP (Batten, 1992; Bovill et al., 2013), including six relative traits for PH (Rph), SN (Rsn), GN (Rgn), TGW (Rtgw), GWP (Rgwp), and StWP (Rstwp).

### Data analysis

Analyses of variance (ANOVA), the least significant difference (LSD) test and simple correlation coefficients (*r*) between different traits were calculated using SPSS 18.0 software (SPSS Inc., Chicago, IL, USA). The adequate model for ANOVA used two factors in a no repeat trial design. All factors involved were considered sources of random effects. Multiple comparison tests for the traits between “treatments” were calculated by taking all of the RILs as replicates and using the average value of the same P conditions for each trait. The broad-sense heritability ($h_{B}^{2}$) was estimated according to the following formula: $h_{B}^{2}=\sigma_{g}^{2}/\left(\sigma_{g}^{2}+\sigma_{e}^{2}\right)$, where $\sigma_{g}^{2}$ was the genotypic variance and $\sigma_{e}^{2}$ was the total error variance; the variance of P concentration was excluded. Spearman's correlation coefficients were calculated for all the traits.

### QTL and meta-QTL analysis

A high-density genetic map for 184 RILs of “TN18 × LM6” (Zhang, 2014, Figure S1) was employed in the QTL analysis. The map comprised of 10,739 loci (5399 unique loci) on all the 21 chromosomes, including 5548 DArTs, 5085 SNPs, and 106 SSRs or EST-SSRs. The total map length was 3,394.47 cM and the density was 0.63 cM/ marker. The Windows QTL Cartographer 2.5 software (http://statgen.ncsu.edu/qtlcart/WQTLCart.htm) was used to perform the QTL mapping, and composite-interval mapping (CIM) was selected to search for QTLs of each trait separately for (i) each of the four environments (Trial 1, Trial 2, Trial 3, and Trial 4), for (ii) the average value of the same P level across different years in the seedling (Trial 1 and Trial 2) and maturity (Trial 3 and Trial 4) stages, and for (iii) the relative traits calculated by dividing the values of LP by the values of NP. The parameter setup “model 6 standard analysis” was used with a walk speed of 0.5 cM; “forward and backward” regression for the selection of the markers was used to control the genetic background, with up to five control markers, and a blocked window size of 10 cM was used to exclude closely linked control markers at the tested site. The threshold for declaring the presence of a significant QTL was defined by 1,000 permutations at *p* ≤ 0.05 (Churchill and Doerge, 1994), and a minimum LOD score of 3.0 was chosen. The LOD threshold value of different trait-treatment combinations varied from 3.20 to 4.03. The confident intervals for a QTL detected in more than one environment (including AV) were confirmed by meta-QTL analysis using Biomercator 2.0 software, and AIC = 4 (model 4) was used in the step Meta-analysis 2/2 (http://www.genoplante.com). We defined a QTL cluster as three or more traits with significant QTLs having overlapping confidence intervals (Stoll et al., 2000; Guo et al., 2012; Kong et al., 2013; Gong et al., 2016), and meta-analysis was also performed on each QTL cluster to determine the confidence interval.

### EST sequences related to QTLs

To find the EST sequences related to QTLs, the sequence of the markers covered by QTLs were obtained by Blastn in the EST data base of NCBI (http://blast.ncbi.nlm.nih.gov/Blast.cgi). If the results of comparison were with E-value less than 1e^−15^, query cover more than 80% and the ident more than 90%, these markers were defined to be highly homologous with EST sequences (Rampant et al., 2011).

## Results

### Phenotypic variation

The parents of the RIL populations, TN18 and LM6, exhibited distinct differences in most of the investigated traits in both the hydroponic culture and field trials (Table S2). Transgressive segregation was observed for all of the 96 trait-treatment combinations. All 24 traits in each trait-treatment exhibited a continuous distribution.

The results of ANOVA, using the average values of the same P treatments for each trait, showed that genotypes and the effects of P levels were significant for all investigated traits at *p* ≤ 0.01 (Table S3). The $h_{B}^{2}$ for the targeted traits ranged from 58.78 (RPutE) to 92.61% (RSDW) and from 51.12 (StPutE) to 86.23% (SL) at the seedling and maturity stages, respectively (Table S3). In general, the four biomass traits (RDW, SDW, TDW, and RSDW) showed higher $h_{B}^{2}$ values (average 92.34%) than seven PE traits (RPC, SPC, TPC, RSPC, RPutE, SPutE, and TPutE) (average 64.59%), and the nine yield traits (PH, GN, SL, FSS, SSS, SN, TGW, GWP, and StWP) had higher $h_{B}^{2}$ values (average 71.49%) than four PE traits (GPC, StPC, GPutE, and StPutE) (average 56.27%).

### Correlation analysis

The correlation coefficients (*r*) among the 11 seedling traits for the average values of four P treatments were almost all significant between biomass traits, between PE traits, and between biomass and PE traits (Table S4-1). In addition, the *r* values among the 13 maturity traits were mostly significant between yield traits and between PE traits (Table S4-2). Between yield and PE traits, five yield traits (SN, PH, TGW, GWP, and StWP) were significant correlated to all four PE traits, and the other four yield traits (SL, FSS, SSS, and GN) were not significant to the PE traits except for SL and StPC as well as GN and GPutE. For correlations between the 13 maturity traits and the 11 seedling traits (Table S4-3), the *r* values were all significant between PH/StWP/StPC and all the seedling traits, and were mostly significant between SL/TGW/GPC/GWP/SN and the seedling traits. However, the *r* values were nearly all not significant between SSS/GUPE and all the seedling traits.

For relative traits, a minority of *r* values were significant and positive (Table S4-4). Between relative traits and PE traits, Rstwp was significant correlated to all the four PE traits, and Rgwp was significant correlated to three of the four PE traits (GPC, GPutE, and StPutE).

### Effects of low P input

The LSD test showed that the average values of the investigated traits were in most cases significantly different between the LP and NP treatments (Table S2). SDW, TDW, SPC, RPC, and TPC were all decreased in parallel with the reduction of P concentration in the nutrient solution. Contrarily, there was an extremely significant increase for RSDW, RSPC, RPutE, SPutE, and TPutE in LP treatments compared with the NP treatments (Table S2). Similarly, PH, SN, TGW, GWP, StWP, GPC, and StPC were mostly significantly decreased in the LP treatments; but extremely significant increases of GPutE and StPutE were found in the LP treatment under Trial 3, while not significant increases were found under Trial 4. Moreover, the differences of four spike traits (SL, FSS, SSS, and GN) were not significant at different P levels. These results showed that the low P input could intensely affect most of the tested traits of wheat.

### Major characteristics of the located QTLs

#### Hydroponic culture trials

For the 11 seedling traits, a total of 55 additive QTLs (68 QTLs for trait-treatment combinations) were detected on 10 chromosomes: 1A, 1D, 3B, 3D, 4B, 4D, 5D, 6A, 6B, and 7B (Table S5, Figure S1). Of these, 21, 17, and 17 QTLs were detected for the biomass traits (RDW, SDW, RSDW, and TDW), PupE traits (RPC, SPC, RSPC, and TPC) and PutE traits (RPutE, SPutE, and TPutE), respectively. For different P levels, 24 and 24 QTLs were identified only in LP and NP treatments, respectively. An individual QTL explained between 4.52 (RSDW) to 50.28% (RSDW) of the phenotypic variation. The highest LOD value for single QTL was 31.55 for *QRsdw-4B*. Among them, 17 QTLs showed positive additive effects with TN18 increasing the effects of QTLs, whereas 38 QTLs had negative additive effects with LM6 increasing the QTL effects. Using the average value (AV) of the same P levels, 39 QTLs were detected (Table S5, Figure S1). Of these, 22 QTLs were found at the same chromosome region as the LP or/and NP treatment(s), one QTL (*QSdw-5D.1*) was detected both in NPAV and LPAV treatments, and seven and nine QTLs were found only in NPAV and LPAV, respectively.

**Figure 1:**
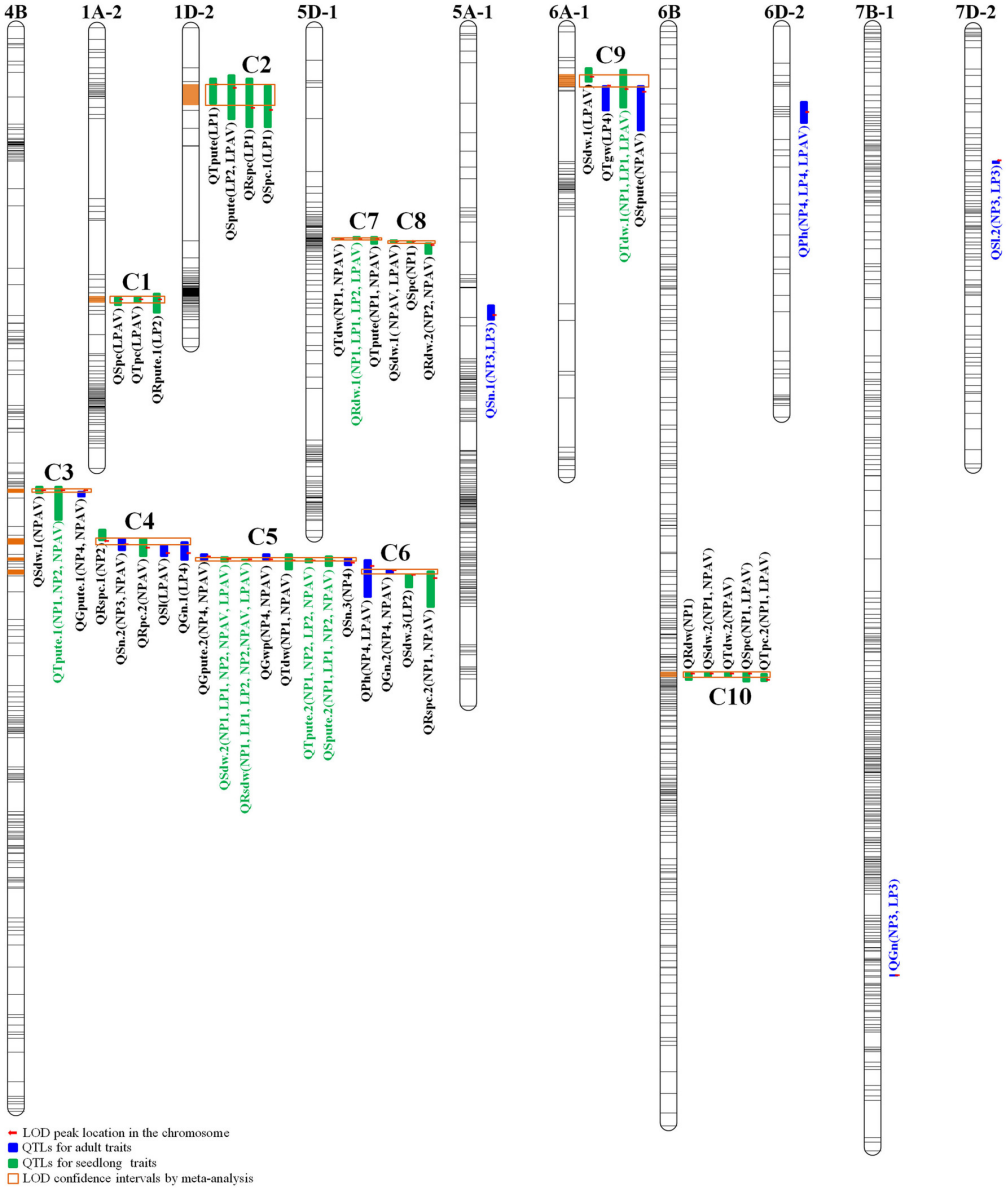
**Locations of QTLs based on RILs derived from TN18 × LM6, including 11 RHF-QTLs and 10 QTL clusters**. QTL intervals were determined by dropping 1 unit in both directions of peak LOD values (1,000 permutation test with *p* ≤ 0.05).

Seven relatively high-frequency QTLs (RHF-QTLs) or relatively stable QTLs (20/68 × 100% = 29.41% QTLs for trait-treatment combinations) were expressed in more than two treatments (Table 3, Figure 1) and included six traits (RDW, SDW, RSDW, TDW, SPutE, and TPutE). Six RHF-QTLs were expressed in LP and NP and AV treatments, but one RHF-QTL (*QTpue-4B.1*) in NP and AV treatments. The additive effects of all the RHF-QTLs, except for *QRsdw-4B*, were negative, showing that the increasing QTL effects came from LM6. Surprisingly, the average *R*^2^ of *QRsdw-4B* was as high as 41.06% (ranged from 26.91 to 50.28%). Hence, this RHF-QTL should be a Mendelian gene. The contributions of *QSpute-4B.2* and *QTpute-4B.2* were 24.87 and 17.68% respectively, indicating they should be major QTLs. Moreover, four RHF-QTLs (*QRdw-5D.1, QSdw-4B.2, QTdw-6A.1*, and *QTpute-4B.1*) had higher contributions.

**Table 3:** **Relatively high-frequency QTLs (RHF-QTLs) detected in more than two treatments under hydroponic culture trials or field trials**.

**Traits**	**QTLs**	**Treatments**	**Marker intervals**	**Additive effects**			***R***^**2**^ **(%)**		
				**Max**	**Min**	**Average**	**Max**	**Min**	**Average**
**HYDROPONIC CULTURE TRIALS**									
RDW	*QRdw-5D.1*	NP1, LP1, LP2, LPAV	*D-2323329-D-3948435*	−0.91	−0.92	−0.91	15.23	10.48	12.85
SDW	*QSdw-4B.2*	NP1, LP1, NP2, NPAV, LPAV	*S-1040960-S-1078626*	−4.18	−6.40	−5.24	19.10	8.71	13.94
RSDW	*QRsdw-4B*	NP1, LP1, NP2, LP2, NPAV, LPAV	*D-3940950-S-3024027*	0.04	0.02	0.03	50.28	26.91	41.06
TDW	*QTdw-6A.1*	NP1, LP1, LPAV	*D-1118135-S-1079131*	−5.03	−10.48	−7.75	14.75	10.17	12.46
SPutE	*QSpute-4B.2*	NP1, LP1, NP2, NPAV	*D-3940950-D-1138250*	−0.76	−1.16	−0.93	30.31	18.75	24.87
TPutE	*QTpute-4B.1*	NP1, NP2, NPAV	*D-1051883-Ku_c63300_1309*	−0.54	−0.74	−0.62	10.51	10.12	10.38
	*QTpute-4B.2*	NP1, NP2, LP2, NPAV	*S-3024027-D-1138250*	−0.67	−1.02	−0.84	23.09	11.33	17.68
**FIELD TRIALS**									
PH	*QPh-6D*	NP4, LP4, LPAV	*D-1246541-D-2265140*	2.07	1.86	1.96	13.49	12.03	12.76
SL	*QSl-7D.2*	NP3, LP3	*wPt-7508-D-3956292*	0.19	0.18	0.18	8.29	7.49	7.89
SN	*QSn-5A.1*	NP3, LP3	*D-1207347-D-1089337*	1.07	0.92	0.99	12.06	11.90	11.98
GN	*QGn-7B*	NP3, LP3	*D-1390136-S-991542*	2.39	1.82	2.11	13.06	10.36	11.71

#### Field trials

A total of 68 additive QTLs (72 QTLs for trait-treatment combinations) for 13 traits were detected on 17 chromosomes except for 1B, 2B, 3D, and 4D (Table S5, Figure S1). Of those, 52, 4, and 12 QTLs were detected for nine yield traits (PH, SN, SL, GN, FSS, SSS, TGW, GWP, and StWP), PupE traits (GPC and StPC) and PutE traits (GPutE and StPutE), respectively. For different P levels, 25 and 39 QTLs were detected only in LP and NP treatments, respectively. An individual QTL could explain the phenotypic variation ranging from 6.41 (*QTgw-6A)* to 23.54% (*QGpute-4B.2*). The highest LOD value for a single QTL was 12.02 for *QGpute-4B.2*. Among them, 40 QTLs showed positive additive effects with TN18 increasing the effects of QTLs, whereas 28 QTLs had negative effects with LM6 increasing the QTL effects. Using the AV of the same P conditions, 39 QTLs were detected (Table S5, Figure S1). Of these, 16 QTLs were at the same chromosome region as LP or/and NP treatment(s), and 12 and 11 QTLs were detected only in NPAV and LPAV, respectively.

Four RHF-QTLs (8/72 × 100% = 11.11% QTLs for trait-treatment combinations) were expressed in more than two treatments (Table 3, Figure 1) and included four traits (PH, SL, SN, and GN), with the average contributions ranging from 7.89 (SL) to 12.76% (PH). All RHF-QTLs were expressed in LP and NP, and one RHF-QTL (*QPh-6D*) was also expressed in AV. Contrary to the RHF-QTLs for seedling traits, the additive effects of all the RHF-QTLs were positive with the increasing effects coming from TN18. The *R*^2^ of three RHF-QTLs, *QPh-6D, QSn-5A.1*, and *QGn-7B* were over 10%, indicating that these were important RHF-QTLs.

#### Relative traits

To better understand the responses of P-deficiency stress, the QTLs for the relative traits were mapped. A total of 15 QTLs were detected on 2A, 2B, 3A, 3B, 3D, 5B, 5D, 6A, 7A, and 7D chromosomes (Table S5, Figure S1). Of these, 1, 9, 2, and 3 QTLs were identified for Rph, Rsn, Rgwp and Rstwp, respectively. An individual QTL explained between 6.71 (*QRsn-7D*) to 13.93% (*QRstwp-2A*) of the phenotype variations, and the *R*^2^ of seven QTLs (*QRsn-2B, QRsn-3B.3, QRsn-3D, QRgwp-3B, QRstwp-2A, QRstwp-5B*, and *QRstwp-7D*) were more than 10%. The highest LOD value for a single QTL was 5.71 for *QRsn-3B.2*. Among them, the additive effects of ten QTLs were positive with TN18 increasing the QTL effects; whereas five QTLs had negative effects with LM6 increasing the QTL effects.

### QTL clusters

Considering the QTLs detected in the AV treatments, a total of 10 QTL clusters (C1-C10) were mapped to six chromosomes (1A, 1D, 4B, 5D, 6A, and 6B) and involved 80 out of the 219 QTLs (36.53%) for trait-treatment combinations (Table 4, Figure 1). These clusters were related to most of the investigated traits except for FSS, SSS, StWP, and StPC. Seven RHF-QTLs were detected in four Clusters: C3, C5, C7, and C9. All these QTL clusters could be classified into two types: detected only on seedling traits (type I, including C1, C2, C7, C8, and C10), and detected simultaneously on seedling and maturity traits (type II, C3-6, and C9).

**Table 4:** **QTL clusters for more than three traits at seedling and maturity stages**.

**Codes/type**	**Chromo-somes**	**Marker intervals**	**No. of QTLs**	**QTLs**	**Treatments^a^**	**Additive effects**	***R^2^* (%)**
C1/I	1A-2	*wPt-667566-swes139*	3	*QSpc*	LPAV	−0.02	7.77
				*QTpc*	LPAV	−0.02	7.42
				*QRpute.1*	LP2	−0.27	11.30
C2/I	1D-2	*D-3959209-D-2251236*	4	*QSpc.1*	LP1	−0.04	11.30
				*QRspc*	LP1	0.05	10.13
				*QSpute*	LP2, LPAV	3.94	10.24
				*QTpute*	LP1	3.53	8.71
C3/II	4B-1	*D-1051883-D-1113185*	3	*QSdw.1*	NPAV	−3.45	7.80
				*QTpute.1*	NP1, NP2, NPAV	−0.62	10.38
				*QGpute.1*	NP4, NPAV	−0.60	9.86
C4/II	4B-1	*D-3022151-D-1040960*	5	*QRspc.1*	NP2	0.01	9.69
				*QSn.2*	NP3, NPAV	−1.11	20.60
				*QSl*	LPAV	0.16	8.39
				*QGn.1*	LP4	2.41	14.89
				*QRpc.2*	NPAV	0.01	14.18
C5/II	4B-1	*D-1083795-D-3940950*	8	*QSdw.2*	NP1, LP1, NP2, NPAV, LPAV	−5.21	13.96
				*QTdw*	NP1, NPAV	−5.64	11.76
				*QRsdw*	NP1, LP1, LP2, NP2, NPAV, LPAV	0.03	41.06
				*QSpute.2*	NP1, LP1, NP2, NPAV	−0.91	25.09
				*QTpute.2*	NP1, NP2, LP2, NPAV	−0.83	18.00
				*QSn.3*	NP4	−1.26	19.20
				*QGwp*	NP4, NPAV	−1.75	11.09
				*QGpute.2*	NP4, NPAV	−1.01	23.53
C6/II	4B-1	*D-4008856-D-1138250*	4	*QGn.2*	NP4, NPAV	1.68	7.08
				*QRspc.2*	NP1, NPAV	0.03	14.19
				*QSdw.3*	LP2	−2.87	8.50
				*QPh*	NP4,LPAV	−2.13	15.27
C7/I	5D-1	*D-2323329-D-3948435*	3	*QRdw.1*	NP1, LP1, LP2, LPAV	−0.82	11.21
				*QTdw*	NP1, NPAV	−5.03	10.27
				*QTpute*	NP1, NPAV	−0.58	7.19
C8/I	5D-1	*D-1055236-D-3956782*	3	*QSdw.1*	NPAV, LPAV	−3.03	7.75
				*QSpc*	NP1	−0.04	8.22
				*QRdw.2*	NP2, NPAV	−0.71	10.89
C9/II	6A-1	*S-1149480-D-1122446*	4	*QSdw*	LPAV	−2.98	9.70
				*QTgw*	LP4	−0.95	6.41
				*QTdw.1*	NP1, LP1, LPAV	−6.40	12.09
				*QStpute*	NPAV	−3.30	9.49
C10/Â-I	6B	*D-3953053-D-991702*	5	*QRdw*	NP1	−0.82	11.59
				*QSdw.2*	NP1, NPAV	−4.66	10.82
				*QSpc*	NP1, LPAV	−0.05	11.52
				*QTdw.2*	NPAV	−4.44	10.48
				*QTpc.2*	NP1, LPAV	−0.05	14.20

## Disussion

### Morphological traits indexes for the evaluation of PE

P supply level has a significant influence on the yield and PupE traits of crops. In this study, the LP input could lead to the reduction of biomass, yield and PupE traits, and the promotion of PutE traits during the whole growth duration of wheat, which is in accordance with previous studies (Su et al., 2006, 2009; Guo et al., 2012; Zhang and Wang, 2015). The PE, which includes the PupE and PutE, must be assessed by measuring P concentration in plant tissue, which is so complicated that it is almost impossible to identify in a large-scale of genotypes such as in breeding programs. Thus, it is necessary to seek some morphological traits instead of using element measurements to reflect the PE indirectly. It has been documented that root architectural traits such as lateral branching and root hair density are clearly advantageous for PE (Lynch, 2007; Ao et al., 2010; Bayuelo-Jiménez et al., 2011; Péret et al., 2011; Niu et al., 2013; Azevedo et al., 2015; Kabir et al., 2015; van de Wiel et al., 2016), however monitoring these traits and using them as selection indexes are time-consuming.

In this study, the correlation analysis demonstrated that the *r* values between biomass traits (SDW, RDW, TDW, and RSDW) and PE traits (SPC, RPC, TPC, RSPC, SPutE, RPutE, and TPutE) were almost all significant, indicating that the biomass traits could be used as the primary criteria for PE (Table S4-1). Similarly, significant and positive correlations were discovered between five yield traits (SN, PH, TGW, GWP, and StWP) and four PE traits (GPC, StPC, GPutE, and StPutE), and between two relative traits (Rstwp and Rgwp) and four PE traits, indicating that these yield traits and relative traits were also able to reflect PE to a certain extent (Table S4-2). In general, the biomass traits, the five yield traits and the two relative traits can be considered as the primary and rapid morphological indexes for the evaluation of PE instead of using element determinations, and the outcomes make it easy to identify PE on a large-scale.

Moreover, the 11 targeted traits at the seedling stage were also significantly correlated to six yield traits (SN, PH, SL, TGW, GWP, and StWP) and two PupE traits (GPC and StPC) (Table S4-3) at the maturity stage, showing that some seedling traits could reflect maturity traits to a certain extent. These results are similar to the conclusions of Ryan et al. (2015) that early vigor can improve the efficiency of P acquisition.

### QTL location

The genetic linkage map we constructed was a high-density map with the average density of 0.63 cM/marker. Using this map, the numbers of markers involved in QTLs were increased and the accuracy of QTLs location was enhanced. A total of 163 QTLs for seedling and maturity traits under different P levels and their AV, and 15 QTLs for relative traits were detected. The average confidence interval was 1.94 cM, and the interval of *QStpute-3B.1* was merely 0.08 cM (Figure S1).

The P treatments can greatly affect the QTLs for P efficiency. For all the 123 QTLs under different P levels, 49 (49/123 × 100% = 39.84%) and 64 (64/123 × 100% = 52.03%) QTLs were detected specifically under LP and NP treatments, respectively. Only 10 RHF-QTLs were detected simultaneously in the two P levels (Table 3). These results indicated that the overwhelming majority of QTLs were inclined to be express in a specific P level. The RHF-QTLs should be the important potential targets for marker-assisted selection (MAS) in wheat breeding programs.

Genetic maps have been used widely for QTL mapping for agronomic traits (Li et al., 2003; Su et al., 2006; Cuthbert et al., 2008; Gegas et al., 2010; Cui et al., 2014; Xu et al., 2014; Zhang et al., 2014), quality traits (Liang et al., 2010; Wang et al., 2011; Deng et al., 2015); fatty acid content in gratin (Wang et al., 2011), and mineral nutrition traits (Fontaine et al., 2009; Peleg et al., 2009; Su et al., 2009; Blair et al., 2010; Guo et al., 2012; Kong et al., 2013; Gong et al., 2016; Hussain et al., 2016; Hitz et al., 2017) in wheat. It allows us to compare our QTL mapping results with the previously mapped QTLs. Some QTLs for P-related and morphological traits in the present study have also been detected in the same or adjacent marker regions of previous QTLs (Table 5). However, the majority of QTLs were mapped in new marker regions in the present study possibly because of the distinct component markers and different genetic background.

**Table 5:** **QTLs detected in the same or adjacent marker regions in this paper and in previous studies**.

**Chromo-somes**	**Markers**	**QTLs in this study**	**QTLs detected in previous studies**	**References**
			**Related traits^a^**	
4B	*wpt-5559*	*QTgw.1*	RL	Zhang et al., 2014
3B	*wPt-3921*	*QStpute.2*	CL, PH	Zhang et al., 2014
		*QRsn.2*	CL, PH	Zhang et al., 2014
			SLPC, RN	Zhang and Wang, 2015
5A	*gwm186*	*QGwp.1*	RSA	Kabir et al., 2015
			PH, HI	Xu et al., 2014
5B	*wPt-4936*	*QGpute*	TKW, KNPS	Cui et al., 2014
7B	*wPt-6156*	*QGn*	SKCE, RKUE	Gong et al., 2015

a *RL, longest root length; CL, coleoptile length; PH, plant height; SLPC, stem and leave phosphorus content; RN, the numbers of axial roots; RSA, root surface area; HI, harvest index; TKW, thousan-kernel weight; KNPS, kernel number per spike; SKCE, shoot K concentration; RKUE, root K utilization efficiency*.

### Import QTL clusters

In wheat, a large number of QTL clusters have been mapped in the same genomic regions (Quarrie et al., 2006; Crossa et al., 2007; Guo et al., 2012; Kong et al., 2013; Zhao et al., 2014; Zhang and Wang, 2015; Gong et al., 2016). Many QTLs are only expressed in a given or a few environment. Therefore, the QTL clusters, which include stable QTL(s) in several environments, should be the most important. In this study, four clusters (C3, C5, C7, and C9) contained RHF-QTLs (Table 4, Figure 1) and were considered as the most important QTL clusters. They were discussed as follows.

Cluster C3 on chromosome 4B of type II (Table 4, Figure 1) involved three QTLs with low contributions at 7.80–10.38%. Of these, one QTL was RHF-QTL (*QTpute-4B.1*); and two QTLs (*QTpute-4B.1* and *QGpute-4B.1*) were detected for PutE traits. Cluster C5 on 4B of type II involved eight QTLs with high contributions at 11.09–41.06%. The C5 included four RHF-QTLs: *QSdw-4B.2, QRsdw-4B, QSpute-4B.2*, and *QTpute-4B.3*. For PutE traits, two RHF-QTLs (*QSpute-4B.2* and *QTpute-4B.3*) and one QTL (*QGpute-4B.2*) were detected, indicating that the C5 was a stable PutE locus. Cluster C7 on 5D were detected at seedling stage (type I) and included three QTLs with contributions of 7.19–11.21%. Of these, one QTL was RHF-QTL (*QRdw-5D.1*); and one QTL (*QTpute-5D*) was detected for PutE trait. Cluster C9 on chromosome 6A of type II included four QTLs with the contributions of 6.41–12.09%. Of these, one QTL was RHF-QTL (*QTdw-6A.1*); and one QTL (*QStpute-5D*) was detected for PutE trait. All the four clusters contained the biomass/yield traits and PutE traits. Except for *QRsdw* in C5, all the QTLs within a cluster of the four QTL clusters had negative additive effects with LM6 increasing the QTL effects and showed a favorable relationship for breeding programs, indicating that the morphological traits and PutE can be simultaneously improved. The markers in these QTL clusters should be useful for MAS in wheat breeding programs of PE.

In addition, we found a QTL cluster (C6) on chromosome 4B (Table 4, Figure 1), which involved a QTL for PH (*QPh-4B*) explaining as high as 15.27% of the phenotypic variation. The additive effect of *QPh-4B* was negative, indicating that the allele of TN18 deceased the PH. On the other hand, TN18 is a semi-dwarf habit with the *Rht-B1b* gene by pedigree analysis. So we conjectured that the *QPh-4B* is *Rht-B1b*. The *Rht-B1b* gene has effects on coleoptile length, plant height (Rebetzke et al., 2014) and root length (Wojciechowski et al., 2009). In this study, the *Rht-B1b* reduced the PH and SDW (biomass traits), but increased the GN (yield traits) and RSPC (PupE traits).

### EST sequences and QTLs

The ESTs associated with important agronomic traits can provide significant information for the functional analysis of complicated quantitative traits (Wang et al., 2011). So far, a few studies have been reported QTLs of complicated agronomic traits linked to EST sequences. In this study, the 178 QTLs we detected covered 283 markers and included 187 DArTs, 87 SNPs, and 9 SSRs. A total of 29 markers were found highly homologous with EST sequences. These ESTs were probably predicted to participate in transcription and translation processes by the NCBI automatic prediction program (Table 6).

**Table 6:** **ESTs and associated QTLs detected in hydroponic culture and fields trials**.

**Marker names**	**Chromo-somes**	**Gnenebank ID**	**Function of ESTs**	**Species**	**Associated QTLs**	**QTL clusters**
*D-1091928*	1D-1	BQ619650.1	cDNA clone TaLr1176A04F	Wheat	*QTgw.1*	
*D-3959209*	1D-2	JZ889824.1	Unknown	Wheat	*QSpc.1, QTpute, QSpute, QRspc*	C2
*D-2251236*	1D-2	EB512014.1	cDNA clone Ta07b_03k13	Wheat	*QSpc.1, QTpute, QSpute, QRspc*	C2
*D-1218473*	1D-2	CA637847.1	cDNA clone wre1n.pk0003.g6	Wheat	*QSpc.2*	
*S-988596*	2A-1	CK200422.1	Unknown	Wheat	*QTgw*	
*S-1248551*	2A-1	HX153097.1	cDNA clone rwhxs2089h22	Wheat,	*QFss*	
*S-2253907*	2A-2	CA686793.1	cDNA clone wlm96.pk034.j5	Wheat	*QGn.2*	
*D-1008756*	3B-1	CJ877191.1	cDNA clone whthls23j04	Wheat, ogihara	*QRdw.1*	
*S-1090569*	3D	EG390843.1	cDNA clone BG01022B2G11.r1	Leymus cinereus × Leymus triticoides	*QRsn*	
*D-1166619*	4A-1	EG388769.1	cDNA clone BG01023A2F09.f1	Maize, rice, wheat	*QRpute*	
*D-1003776*	4B-1	CJ815588.1	cDNA clone whsct26j05	Wheat, ogihara	*QSpue.1*	
*D-1051883*	4B-1	BG605126.1	cDNA clone WHE2327_F04_L07	Wheat	*QSdw.1, QTpute.1, QGpute.1*	C3
*S-2275640*	4B-1	CN013155.1	cDNA clone WHE3957_D08_H15	Wheat	*QStpc*	
*S-3941408*	4B-1	JZ889353.1	Unknown	Wheat	*QGn.2, QPh, QSdw.3, QRspc.2*	C6
*S-2282007*	5B-1	BJ255428.1	cDNA clone whf23l11	Wheat, ogihara	*QStwp.1*	
*S-989092*	5B-2	CJ530625.1	cDNA clone rwhec15i11	Wheat	*QPh*	
*S-1055033*	5D-1	BM817221.1	Hypothetical protein	Barley	*QRpc.1, QSpute, QSss*	
*D-1117581*	5D-1	CJ845927.1	cDNA clone whatlal39m10	Wheat, ogihara	*QSdw.1, QSpc, QRdw.2*	C8
*D-1261108*	5D-1	AL808725.1	cDNA clone B03_A22_plate_7	Wheat	*QRpc.2, QSdw.2*	
*S-3024384*	5D-2	DR741364.1	Unknown	Wheat	*QRph*	
*S-1079131*	6A-1	DR741550.1	Unknown	Wheat, canola	*QTdw.1, QTgw, QStpute, QSdw.1*	C9
*D-1722489*	6A-3	CA643588.1	cDNA clone wre1n.pk0066.h11	Wheat	*QRspc*	
*D-1106806*	6B	CJ681614.1	cDNA clone whok5b08	Wheat, ogihara	*QTpc.1*	
*S-985767*	6B	BJ274667.1	cDNA clone whoh4c23	Wheat, ogihara	*QSdw.1, QTdw.1*	
*D-3953053*	6B	CJ530191.1	cDNA clone rwhec13o24	Wheat, ogihara	*QTpc.2, QRdw, QTdw.2, QSdw.2, QSpc*	C10
*D-1095545*	6B	HX098758.1	cDNA clone whxp1006k03	Wheat	*QRpue, QStpute*	
*D-2265140*	6D-2	BU100490.1	cDNA clone WHE3354_A01_B02	Wheat	*QPh*	
*D-1390136*	7B-1	BJ208855.1	cDNA clone wh11e08	Wheat, ogihara	*QGn*	
*S-989952*	7D-2	CK208711.1	Unknown	Wheat, canola	*QFss.4, QSl.3*	

A total of 51 QTLs detected in our study were likely to huddle together around the ESTs (Table 6). Of these, 19 QTLs were detected specifically under LP treatment and 16 QTLs were detected specially under NP treatment. Four RHF-QTLs (*QTpute-4B.1, QTdw-6A.1, QPh-6D*, and *QGn-7B*) and six QTL clusters (C2, C3, C6, C8, C9, and C10) were found linked to ESTs possibly. The metabolic functions of some ESTs were annotated. For example, *QRpc-5D.1, QSpute-5D*, and *QSss-5D* around *S-1055033* was highly homologous with the EST sequence of BM817221.1, which encoded hypothetical protein F11C1.220 that was related to drought- and salt-stressed in barley (Ozturk et al., 2002). *QTdw-6B.1* and *QSdw-6B.1* gathered around *S-985767* was highly homologous to EST sequences of CJ530191.1, which probably played a part in two storage-protein gene families of wheat and ogihara (Kawaura and Ogihara, 2006). Although we cannot currently illuminate the mechanisms between the functions of the ESTs and the QTLs for PE and morphological traits, it is important that the QTLs may be nestled with ESTs. These results may provide a molecular foundation for annotation of QTLs and QTL clusters. The relationship between these ESTs and QTLs is worth studying in the future.

## Author contributions

Designed the experiments: SL and FK. Performed the experiments: YY, MZ, HZ, XZ, YG, and YZ. Analyzed the data: YY and MG. Wrote the paper: YY and SL.

### Conflict of interest statement

The authors declare that the research was conducted in the absence of any commercial or financial relationships that could be construed as a potential conflict of interest.

## Abbreviations

EST: Expressed sequence tag
FSS: Fertile spikelet number per spike
GN: Grain number per spike
GPC: Grain P-content per plant
GPutE: Grain P-utilization efficiency
GWP: Grain weight per plant
MAS: Marker-assisted selection
PE: Phosphorus efficiency
PH: Plant height
PupE: Phosphorus uptake efficiency
PutE: Phosphorus utilization efficiency
QTL: Quantitative trait locus
RDW: Root dry weight per plant
Rgn: Relative trait for GN
Rgwp: Relative trait for GWP
RHF-QTL: Relatively high-frequency QTL
RIL: Recombinant inbred line
RPC: Root P-content per plant
Rph: Relative trait for PH
RPutE: Root P-utilization efficiency
RSDW: Ratio of root and shoot dry weight
Rsn: Relative trait for SN
RSPC: Ratio of root and shoot content
Rstwp: Relativetrait for StWP
Rtgw: Relative trait for TGW
SDW: Shoot dry weight per plant
SL: Spike length
SN: Spike number per plant
SPC: Shoot P-content per plant
SPutE: Shoot P-utilization efficiency
SSS: Sterile spikelet number per spike
StPC: Straw P-content per plant
StPutE: Straw P-utilization efficiency
StWP: Straw weight per plant
TDW: Total dry weight per plant
TGW: Thousand grain weight
TPC: Total P-content per plant
TPutE: Total P-utilization efficiency.
