# Supplementary material for: QTL Mapping for Phosphorus Efficiency and Morphological Traits at Seedling and Maturity Stages in Wheat
Source: Front Plant Sci. 2017 Apr 24;8:614. doi: 10.3389/fpls.2017.00614 (PMC5402226; doi:10.3389/fpls.2017.00614)
Supplement: Supplementary file 1 [file Table1.DOCX]

**Table S1** Nutrient solution ingredients for wheat seedling growth

| Ingredients | Concentration  (mmol/L) | Ingredient | Concentration  (μmol/L) |
| --- | --- | --- | --- |
| (NH_4_)_2_SO_4_·H_2_O | 1 | H_3_BO_3_ | 1 |
| Ca(NO_3_)_2_·4H_2_O | 1 | CuSO_4_·5H_2_O | 0.5 |
| KH_2_PO_4_ | 0.2 | ZnSO_4_·7H_2_O | 1 |
| KCl | 1.8 | MnSO_4_·H_2_O | 1 |
| MgSO_4_·7H_2_O | 0.5 | FeEDTA | 100 |
| CaCl_2_ | 1.5 | (NH_4_)_6_Mo_7_O_24_·4H_2_O | 0.1 |
