# Supplementary material for: QTL Mapping for Phosphorus Efficiency and Morphological Traits at Seedling and Maturity Stages in Wheat
Source: Front Plant Sci. 2017 Apr 24;8:614. doi: 10.3389/fpls.2017.00614 (PMC5402226; doi:10.3389/fpls.2017.00614)
Supplement: Supplementary file 2 [file Table2.DOCX]

**Table S2** Phenotypic performance of the RILs and their parents under hydroponic culture and field trials

| Traits | Treatments | Parents | |  | RILs (n=184) | | | | | |
| --- | --- | --- | --- | --- | --- | --- | --- | --- | --- | --- |
|  |  | TN18 | LM6 |  | Max | Min | Average^a^ |  | SD | CV (%)^b^ |
| ***Hydroponic culture trials*** | | |  |  |  |  |  |  |  |  |
| SDW | NP1 | 60.50 | 54.38 |  | 109.88 | 22.83 | 55.91 | c | 13.77 | 24.62 |
| (mg·plant^-1^) | LP1 | 55.13 | 38.88 |  | 93.63 | 25.33 | 52.31 | d | 12.43 | 23.77 |
|  | NP2 | 87.88 | 99.33 |  | 104.25 | 38.38 | 69.52 | a | 13.35 | 19.21 |
|  | LP2 | 49.13 | 47.88 |  | 82.88 | 31.00 | 53.90 | cd | 9.37 | 17.38 |
|  | NPAV | 74.19 | 76.85 |  | 107.06 | 30.60 | 62.72 | b | 15.16 | 24.17 |
|  | LPAV | 52.13 | 43.38 |  | 88.25 | 28.17 | 53.11 | d | 11.02 | 20.75 |
| RDW | NP1 | 16.00 | 15.38 |  | 22.13 | 7.00 | 11.92 | de | 2.22 | 18.65 |
| (mg·plant^-1^) | LP1 | 15.88 | 12.00 |  | 19.63 | 6.00 | 11.88 | e | 2.43 | 20.42 |
|  | NP2 | 16.88 | 17.25 |  | 19.50 | 7.75 | 12.85 | bc | 2.26 | 17.60 |
|  | LP2 | 17.63 | 23.00 |  | 22.63 | 8.17 | 14.65 | a | 2.74 | 18.68 |
|  | NPAV | 16.44 | 16.31 |  | 20.81 | 7.38 | 12.39 | cd | 2.34 | 18.87 |
|  | LPAV | 16.75 | 17.50 |  | 21.13 | 7.08 | 13.26 | b | 2.84 | 21.41 |
| TDW | NP1 | 76.50 | 69.75 |  | 132.00 | 34.33 | 67.89 | c | 15.36 | 22.62 |
| (mg·plant^-1^) | LP1 | 71.00 | 50.88 |  | 113.25 | 34.46 | 64.23 | d | 13.90 | 21.64 |
|  | NP2 | 104.75 | 116.58 |  | 122.50 | 48.50 | 82.37 | a | 15.04 | 18.25 |
|  | LP2 | 66.75 | 70.88 |  | 104.38 | 40.38 | 68.55 | c | 11.63 | 16.96 |
|  | NPAV | 90.63 | 93.17 |  | 127.25 | 41.42 | 75.13 | b | 16.82 | 22.39 |
|  | LPAV | 68.88 | 60.88 |  | 108.81 | 37.42 | 66.39 | cd | 12.98 | 19.55 |
| RSDW | NP1 | 0.26 | 0.28 |  | 0.50 | 0.14 | 0.22 | d | 0.05 | 22.87 |
|  | LP1 | 0.29 | 0.31 |  | 0.44 | 0.12 | 0.24 | c | 0.05 | 21.87 |
|  | NP2 | 0.19 | 0.17 |  | 0.28 | 0.11 | 0.19 | f | 0.03 | 14.66 |
|  | LP2 | 0.36 | 0.48 |  | 0.38 | 0.19 | 0.27 | a | 0.03 | 12.27 |
|  | NPAV | 0.23 | 0.23 |  | 0.39 | 0.12 | 0.20 | e | 0.04 | 21.62 |
|  | LPAV | 0.32 | 0.39 |  | 0.41 | 0.16 | 0.25 | b | 0.05 | 18.60 |
| SPC | NP1 | 0.45 | 0.46 |  | 1.15 | 0.25 | 0.54 | c | 0.14 | 26.73 |
| (mg·plant^-1^) | LP1 | 0.17 | 0.12 |  | 0.47 | 0.04 | 0.19 | f | 0.09 | 47.77 |
|  | NP2 | 1.23 | 1.49 |  | 1.74 | 0.45 | 0.95 | a | 0.21 | 22.52 |
|  | LP2 | 0.34 | 0.38 |  | 0.82 | 0.19 | 0.46 | d | 0.12 | 25.05 |
|  | NPAV | 0.84 | 0.97 |  | 1.45 | 0.35 | 0.74 | b | 0.27 | 36.97 |
|  | LPAV | 0.25 | 0.25 |  | 0.64 | 0.12 | 0.33 | e | 0.17 | 52.99 |
| RPC | NP1 | 0.11 | 0.12 |  | 0.19 | 0.03 | 0.10 | c | 0.03 | 31.62 |
| (mg·plant^-1^) | LP1 | 0.03 | 0.02 |  | 0.07 | 0.01 | 0.03 | f | 0.01 | 32.87 |
|  | NP2 | 0.15 | 0.21 |  | 0.22 | 0.04 | 0.12 | a | 0.03 | 26.75 |
|  | LP2 | 0.16 | 0.18 |  | 0.20 | 0.03 | 0.10 | d | 0.03 | 32.64 |
|  | NPAV | 0.13 | 0.17 |  | 0.21 | 0.03 | 0.11 | b | 0.03 | 30.76 |
|  | LPAV | 0.10 | 0.10 |  | 0.13 | 0.02 | 0.07 | e | 0.04 | 61.36 |

^a^ Means in the same column followed by the same letter do not differ significantly according to the LSD test (letter indicating *p* ≤ 0.05);

^b^ CV (%) = SD/average × 100%.

**Table S2** Continued-1

| Traits | Treatments | Parents | |  | RILs (n=184) | | | | | |
| --- | --- | --- | --- | --- | --- | --- | --- | --- | --- | --- |
|  |  | TN18 | LM6 |  | Max | Min | Average |  | SD | CV (%) |
| TPC | NP1 | 0.56 | 0.58 |  | 1.34 | 0.32 | 0.63 | c | 0.16 | 24.65 |
| (mg·plant^-1^) | LP1 | 0.20 | 0.14 |  | 0.51 | 0.07 | 0.22 | f | 0.09 | 41.21 |
|  | NP2 | 1.39 | 1.70 |  | 1.95 | 0.55 | 1.07 | a | 0.25 | 23.16 |
|  | LP2 | 0.50 | 0.56 |  | 0.98 | 0.25 | 0.57 | d | 0.15 | 25.74 |
|  | NPAV | 0.97 | 1.14 |  | 1.65 | 0.44 | 0.85 | b | 0.30 | 35.03 |
|  | LPAV | 0.35 | 0.35 |  | 0.75 | 0.16 | 0.39 | e | 0.21 | 53.07 |
| RSPC | NP1 | 0.24 | 0.27 |  | 0.48 | 0.06 | 0.19 | c | 0.07 | 35.91 |
|  | LP1 | 0.19 | 0.16 |  | 0.85 | 0.02 | 0.22 | b | 0.13 | 57.62 |
|  | NP2 | 0.12 | 0.14 |  | 0.23 | 0.03 | 0.13 | e | 0.03 | 22.45 |
|  | LP2 | 0.48 | 0.48 |  | 0.48 | 0.06 | 0.23 | a | 0.08 | 36.71 |
|  | NPAV | 0.18 | 0.21 |  | 0.35 | 0.05 | 0.16 | d | 0.06 | 37.93 |
|  | LPAV | 0.33 | 0.32 |  | 0.66 | 0.04 | 0.22 | a | 0.11 | 48.08 |
| SPutE | NP1 | 8.13 | 6.48 |  | 14.48 | 2.11 | 5.98 | cd | 1.95 | 32.55 |
| [mg·(μg·mg^-1^)^-1^] | LP1 | 17.67 | 12.68 |  | 78.63 | 5.86 | 18.08 | a | 11.28 | 62.38 |
|  | NP2 | 6.26 | 6.62 |  | 10.59 | 2.14 | 5.24 | e | 1.44 | 27.51 |
|  | LP2 | 7.19 | 6.05 |  | 13.66 | 3.27 | 6.49 | c | 1.79 | 27.64 |
|  | NPAV | 7.19 | 6.55 |  | 12.54 | 2.13 | 5.61 | de | 1.75 | 31.19 |
|  | LPAV | 12.43 | 9.36 |  | 46.14 | 4.56 | 12.28 | b | 9.94 | 80.89 |
| RPutE | NP1 | 2.38 | 1.89 |  | 3.66 | 0.60 | 1.58 | d | 0.53 | 33.38 |
| [mg·(μg·mg^-1^)^-1^] | LP1 | 7.87 | 7.43 |  | 22.18 | 1.37 | 4.71 | a | 2.27 | 48.21 |
|  | NP2 | 1.85 | 1.43 |  | 6.81 | 0.73 | 1.44 | e | 0.57 | 39.85 |
|  | LP2 | 1.94 | 2.90 |  | 7.28 | 1.14 | 2.22 | c | 0.73 | 32.73 |
|  | NPAV | 2.12 | 1.66 |  | 5.23 | 0.66 | 1.51 | de | 0.55 | 36.73 |
|  | LPAV | 4.90 | 5.17 |  | 14.73 | 1.25 | 3.46 | b | 2.11 | 60.86 |
| TPutE | NP1 | 10.49 | 8.37 |  | 14.31 | 3.65 | 7.43 | d | 2.15 | 28.88 |
| [mg·(μg·mg^-1^)^-1^] | LP1 | 24.71 | 18.68 |  | 64.70 | 8.63 | 21.68 | a | 10.88 | 50.17 |
|  | NP2 | 7.91 | 8.00 |  | 12.14 | 2.86 | 6.50 | e | 1.70 | 26.19 |
|  | LP2 | 8.98 | 8.96 |  | 15.95 | 5.00 | 8.46 | c | 2.16 | 25.49 |
|  | NPAV | 9.20 | 8.19 |  | 13.22 | 3.25 | 6.96 | de | 1.99 | 28.63 |
|  | LPAV | 16.85 | 13.82 |  | 40.32 | 6.81 | 15.07 | b | 10.29 | 68.30 |
| ***Field trials*** | |  |  |  |  |  |  |  |  |  |
| PH | NP3 | 60.99 | 51.93 |  | 65.90 | 47.13 | 55.35 | d | 3.73 | 6.74 |
| (cm) | LP3 | 55.30 | 52.27 |  | 64.60 | 45.70 | 53.79 | e | 3.70 | 6.89 |
|  | NP4 | 59.04 | 67.14 |  | 81.62 | 47.90 | 60.84 | a | 5.14 | 8.45 |
|  | LP4 | 62.50 | 71.17 |  | 76.38 | 45.46 | 60.13 | a | 5.36 | 8.92 |
|  | NPAV | 61.74 | 59.53 |  | 70.00 | 48.79 | 58.11 | b | 3.81 | 6.57 |
|  | LPAV | 57.17 | 61.72 |  | 70.45 | 47.66 | 56.98 | c | 3.82 | 6.70 |
| GN | NP3 | 53.28 | 54.60 |  | 71.70 | 39.25 | 58.68 | a | 4.70 | 8.01 |
|  | LP3 | 51.50 | 61.00 |  | 77.44 | 46.80 | 58.11 | ab | 4.96 | 8.54 |
|  | NP4 | 59.00 | 57.00 |  | 73.80 | 41.60 | 56.23 | c | 5.80 | 10.31 |

**Table S2** Continued-2

| Traits | Treatments | Parents | |  | RILs (n=184) | | | | | |
| --- | --- | --- | --- | --- | --- | --- | --- | --- | --- | --- |
|  |  | TN18 | LM6 |  | Max | Min | Average |  | SD | CV (%) |
| GN | LP4 | 68.00 | 69.67 |  | 74.80 | 43.20 | 56.35 | c | 6.16 | 10.93 |
|  | NPAV | 56.14 | 55.80 |  | 68.60 | 45.62 | 57.45 | b | 4.10 | 7.13 |
|  | LPAV | 59.75 | 65.34 |  | 71.18 | 45.10 | 57.24 | bc | 4.38 | 7.65 |
| SN | NP3 | 12.85 | 11.03 |  | 18.88 | 6.18 | 12.18 | a | 3.18 | 26.09 |
|  | LP3 | 8.10 | 8.00 |  | 17.43 | 5.60 | 10.63 | b | 2.55 | 23.96 |
|  | NP4 | 3.20 | 6.60 |  | 15.80 | 1.40 | 8.68 | d | 2.73 | 31.42 |
|  | LP4 | 4.67 | 7.33 |  | 19.00 | 2.60 | 7.96 | e | 2.66 | 33.36 |
|  | NPAV | 8.03 | 8.81 |  | 16.41 | 4.59 | 10.38 | b | 2.24 | 21.58 |
|  | LPAV | 6.39 | 7.67 |  | 14.54 | 5.00 | 9.30 | c | 1.92 | 20.69 |
| SL | NP3 | 8.53 | 7.44 |  | 10.60 | 6.55 | 8.83 | a | 0.65 | 7.32 |
| (cm) | LP3 | 7.85 | 7.30 |  | 10.63 | 7.10 | 8.74 | a | 0.64 | 7.34 |
|  | NP4 | 8.24 | 9.44 |  | 10.94 | 7.00 | 8.26 | c | 0.64 | 7.78 |
|  | LP4 | 8.90 | 9.13 |  | 10.22 | 6.71 | 8.31 | c | 0.66 | 7.91 |
|  | NPAV | 8.39 | 8.44 |  | 10.08 | 7.23 | 8.55 | b | 0.53 | 6.16 |
|  | LPAV | 8.38 | 8.22 |  | 10.43 | 7.42 | 8.53 | b | 0.55 | 6.39 |
| FSS | NP3 | 17.63 | 17.48 |  | 20.63 | 15.00 | 18.15 | bc | 0.85 | 4.68 |
|  | LP3 | 16.80 | 17.00 |  | 20.25 | 15.86 | 18.04 | dc | 0.85 | 4.73 |
|  | NP4 | 18.40 | 18.60 |  | 27.00 | 16.20 | 18.34 | ab | 1.21 | 6.57 |
|  | LP4 | 20.00 | 20.33 |  | 21.00 | 15.40 | 18.48 | a | 1.00 | 5.43 |
|  | NPAV | 18.01 | 18.04 |  | 23.05 | 16.25 | 18.26 | b | 0.81 | 4.45 |
|  | LPAV | 18.40 | 18.67 |  | 20.17 | 15.88 | 18.24 | b | 0.74 | 4.04 |
| SSS | NP3 | 0.00 | 0.00 |  | 1.08 | 0.00 | 0.18 | a | 0.24 | 133.58 |
|  | LP3 | 0.00 | 0.00 |  | 1.60 | 0.00 | 0.18 | a | 0.27 | 148.50 |
|  | NP4 | 0.00 | 1.00 |  | 1.20 | 0.00 | 0.22 | a | 0.27 | 122.88 |
|  | LP4 | 0.00 | 0.00 |  | 1.40 | 0.00 | 0.19 | a | 0.27 | 144.27 |
|  | NPAV | 0.00 | 0.50 |  | 0.73 | 0.00 | 0.20 | a | 0.19 | 94.68 |
|  | LPAV | 0.00 | 0.00 |  | 0.90 | 0.00 | 0.19 | a | 0.18 | 97.06 |
| TGW | NP3 | 48.50 | 49.23 |  | 55.03 | 38.17 | 46.64 | d | 3.53 | 7.57 |
| (g) | LP3 | 42.80 | 47.43 |  | 54.43 | 35.93 | 46.35 | d | 3.47 | 7.48 |
|  | NP4 | 48.20 | 49.27 |  | 62.43 | 42.57 | 53.13 | a | 6.65 | 12.51 |
|  | LP4 | 43.70 | 47.50 |  | 61.93 | 42.57 | 50.42 | b | 3.70 | 7.34 |
|  | NPAV | 48.35 | 49.25 |  | 58.17 | 42.22 | 49.89 | b | 2.77 | 5.56 |
|  | LPAV | 43.25 | 47.47 |  | 58.18 | 39.47 | 48.42 | c | 3.05 | 6.30 |
| GWP | NP3 | 19.67 | 16.97 |  | 36.10 | 9.13 | 19.52 | c | 4.13 | 21.16 |
| (g·plant^-1^) | LP3 | 12.50 | 14.03 |  | 39.17 | 6.31 | 19.14 | c | 5.78 | 30.18 |
|  | NP4 | 19.50 | 28.00 |  | 43.25 | 7.60 | 23.42 | a | 6.04 | 25.76 |
|  | LP4 | 13.50 | 16.00 |  | 48.00 | 3.25 | 17.95 | d | 6.98 | 38.87 |
|  | NPAV | 19.59 | 22.49 |  | 32.72 | 8.37 | 21.33 | b | 4.08 | 19.11 |
|  | LPAV | 13.00 | 15.02 |  | 31.76 | 9.19 | 18.61 | cd | 4.59 | 24.68 |

**Table S2** Continued-3

| Trait^s^ | Treatments | Parents | |  | RILs (n=184) | | | | | |
| --- | --- | --- | --- | --- | --- | --- | --- | --- | --- | --- |
|  |  | TN18 | LM6 |  | Max | Min | Average |  | SD | CV (%) |
| StWP | NP3 | 23.45 | 18.85 |  | 39.94 | 8.93 | 21.56 | c | 5.50 | 25.52 |
| (g·plant^-1^) | LP3 | 13.30 | 15.72 |  | 38.95 | 7.08 | 19.19 | d | 6.01 | 31.31 |
|  | NP4 | 30.25 | 48.50 |  | 75.70 | 12.50 | 32.58 | a | 10.34 | 31.75 |
|  | LP4 | 28.00 | 44.30 |  | 72.38 | 4.88 | 26.38 | b | 10.56 | 40.02 |
|  | NPAV | 26.85 | 33.68 |  | 46.92 | 14.41 | 26.87 | b | 5.98 | 22.26 |
|  | LPAV | 20.65 | 30.01 |  | 44.50 | 7.83 | 22.61 | c | 6.19 | 27.37 |
| GPC | NP3 | 79.61 | 92.30 |  | 172.80 | 24.26 | 72.19 | c | 38.46 | 53.28 |
| (mg·plant^-1^) | LP3 | 37.51 | 84.20 |  | 131.07 | 19.03 | 64.76 | d | 23.20 | 35.83 |
|  | NP4 | 68.53 | 105.40 |  | 170.06 | 25.08 | 97.41 | a | 27.54 | 26.22 |
|  | LP4 | 40.78 | 56.99 |  | 124.09 | 14.90 | 57.28 | e | 23.25 | 40.59 |
|  | NPAV | 74.07 | 98.85 |  | 170.06 | 24.67 | 88.02 | b | 23.34 | 26.52 |
|  | LPAV | 39.15 | 70.59 |  | 106.63 | 25.31 | 61.15 | d | 17.48 | 28.59 |
| StPC | NP3 | 24.71 | 17.01 |  | 53.83 | 4.11 | 22.16 | c | 9.83 | 44.35 |
| (mg·plant^-1^) | LP3 | 3.99 | 9.43 |  | 38.23 | 2.51 | 11.76 | e | 5.75 | 48.87 |
|  | NP4 | 40.87 | 93.34 |  | 92.07 | 8.91 | 35.88 | a | 16.68 | 46.48 |
|  | LP4 | 33.23 | 31.33 |  | 59.85 | 3.90 | 22.48 | c | 12.26 | 54.52 |
|  | NPAV | 32.79 | 55.17 |  | 56.83 | 11.26 | 28.79 | b | 9.78 | 33.98 |
|  | LPAV | 18.61 | 20.38 |  | 48.60 | 5.49 | 17.02 | d | 7.15 | 42.01 |
| GPutE | NP3 | 4.86 | 3.12 |  | 12.86 | 2.61 | 5.35 | b | 2.90 | 54.27 |
| [g·(mg.g^-1^)^-1^] | LP3 | 4.17 | 2.34 |  | 25.15 | 1.94 | 6.02 | a | 2.86 | 47.45 |
|  | NP4 | 5.55 | 7.44 |  | 11.87 | 1.81 | 5.66 | ab | 1.76 | 31.12 |
|  | LP4 | 4.47 | 4.49 |  | 18.57 | 1.21 | 5.81 | ab | 2.49 | 42.81 |
|  | NPAV | 5.21 | 5.28 |  | 11.00 | 2.04 | 5.55 | b | 1.52 | 27.39 |
|  | LPAV | 4.32 | 3.42 |  | 16.59 | 2.72 | 6.03 | a | 2.06 | 34.18 |
| StPutE | NP3 | 22.26 | 20.90 |  | 61.44 | 6.31 | 23.76 | c | 10.88 | 45.77 |
| [g·(mg.g^-1^)^-1^] | LP3 | 44.32 | 26.20 |  | 131.87 | 8.23 | 35.66 | a | 19.79 | 55.50 |
|  | NP4 | 22.39 | 25.20 |  | 96.66 | 8.53 | 32.81 | a | 15.88 | 48.41 |
|  | LP4 | 23.59 | 62.64 |  | 126.50 | 5.00 | 35.59 | a | 19.81 | 61.46 |
|  | NPAV | 22.33 | 23.05 |  | 84.96 | 10.14 | 28.40 | b | 10.50 | 36.97 |
|  | LPAV | 33.96 | 44.42 |  | 97.53 | 8.31 | 35.29 | a | 14.15 | 40.09 |
| ***Relative traits*** |  |  |  |  |  |  |  |  |  |  |
| Rph | Trial3 | 0.91 | 1.01 |  | 1.09 | 0.81 | 0.97 | c | 0.05 | 5.37 |
|  | Trial4 | 1.06 | 1.06 |  | 1.32 | 0.75 | 0.99 | a | 0.08 | 8.34 |
|  | AV | 0.98 | 1.03 |  | 1.21 | 0.81 | 0.98 | b | 0.05 | 4.94 |
| Rsn | Trial3 | 0.63 | 0.73 |  | 1.43 | 0.46 | 0.89 | c | 0.22 | 24.38 |
|  | Trial4 | 1.46 | 1.11 |  | 6.29 | 0.28 | 0.98 | a | 0.25 | 25.51 |
|  | AV | 1.04 | 0.92 |  | 3.53 | 0.53 | 0.96 | b | 0.23 | 23.96 |
| Rgn | \| Trial3 \| \| --- \| \| Trial4 \| \| AV \| | 0.97 | 1.12 |  | 1.29 | 0.82 | 0.99 | c | 0.07 | 7.35 |
|  | \| Trial4 \| \| --- \| \| Trial4 \| \| AV \| | 1.15 | 1.22 |  | 1.33 | 0.59 | 1.01 | a | 0.12 | 11.96 |
|  | \| AV \| \| --- \| \| Trial4 \| \| AV \| | 1.06 | 1.17 |  | 1.21 | 0.75 | 1.00 | b | 0.07 | 7.16 |
| Rtgw | Trial3 | 0.88 | 0.96 |  | 1.21 | 0.73 | 0.99 | a | 0.07 | 7.26 |

**Table S2** Continued-4

| Traits | Treatments | Parents | |  | RILs (n=184) | | | | | |
| --- | --- | --- | --- | --- | --- | --- | --- | --- | --- | --- |
|  |  | TN18 | LM6 |  | Max | Min | Average |  | SD | CV (%) |
|  | \| \| Trial4 \| \| --- \| \| Trial4 \| \| AV \| \| \| --- \| --- \| --- \| --- \| \| \| AV \| \| --- \| \| Trial4 \| \| AV \| \| | 0.91 | 0.96 |  | 1.35 | 0.78 | 0.95 | c | 0.12 | 12.93 |
|  | \| \| AV \| \| --- \| \| Trial4 \| \| AV \| \| \| --- \| --- \| --- \| --- \| \| \| AV \| \| --- \| \| Trial4 \| \| AV \| \| | 0.89 | 0.96 |  | 1.17 | 0.83 | 0.97 | b | 0.05 | 5.30 |
| Rgwp | Trial3 | 0.64 | 0.83 |  | 2.03 | 0.24 | 0.98 | a | 0.33 | 33.67 |
|  | \| \| Trial4 \| \| --- \| \| Trial4 \| \| AV \| \| \| --- \| --- \| --- \| --- \| \| \| AV \| \| --- \| \| Trial4 \| \| AV \| \| | 0.69 | 0.57 |  | 2.42 | 0.17 | 0.81 | c | 0.40 | 49.42 |
|  | \| \| AV \| \| --- \| \| Trial4 \| \| AV \| \| \| --- \| --- \| --- \| --- \| \| \| AV \| \| --- \| \| Trial4 \| \| AV \| \| | 0.66 | 0.70 |  | 2.03 | 0.31 | 0.92 | b | 0.29 | 30.88 |
| Rstwp | Trial3 | 0.57 | 0.83 |  | 2.50 | 0.22 | 0.95 | a | 0.37 | 38.76 |
|  | \| \| Trial4 \| \| --- \| \| Trial4 \| \| AV \| \| \| --- \| --- \| --- \| --- \| \| \| AV \| \| --- \| \| Trial4 \| \| AV \| \| | 0.93 | 0.91 |  | 2.43 | 0.13 | 0.87 | c | 0.45 | 51.67 |
|  | AV | 0.75 | 0.87 |  | 1.74 | 0.30 | 0.91 | b | 0.27 | 29.30 |
