# Supplementary material for: QTL Mapping for Phosphorus Efficiency and Morphological Traits at Seedling and Maturity Stages in Wheat
Source: Front Plant Sci. 2017 Apr 24;8:614. doi: 10.3389/fpls.2017.00614 (PMC5402226; doi:10.3389/fpls.2017.00614)
Supplement: Supplementary file 3 [file Table3.DOCX]

**Table S3** Analysis of variance (ANOVA) and the heritability for the investigated traits under hydroponic culture and field trials

| Hydroponic culture trials | | |  |  | Field trials | | | |
| --- | --- | --- | --- | --- | --- | --- | --- | --- |
| Traits | Source of variation | | Heritability |  | Traits | Source of variation | | Heritability |
|  | Genotypes | Treatments | （*h_B_*^2^）% |  |  | Genotypes | Treatments | （*h_B_*^2^）% |
| SDW | 11.30^***^ | 453.21^***^ | 91.87 |  | PH | 5.86^***^ | 23.50^***^ | 85.42 |
| RDW | 12.40^***^ | 106.18^***^ | 92.54 |  | GN | 3.28^***^ | 9.61^***^ | 76.65 |
| TDW | 12.04^***^ | 309.30^***^ | 92.33 |  | SL | 6.26^***^ | 7.30^***^ | 86.23 |
| RSDW | 12.53^***^ | 1099.96^***^ | 92.61 |  | FSS | 2.81^***^ | 10.02^***^ | 73.77 |
| SPC | 1.88^***^ | 2013.93^***^ | 65.30 |  | SSS | 1.31^**^ | 6.07^***^ | 56.65 |
| RPC | 3.18^***^ | 758.08^***^ | 76.06 |  | SN | 2.77^***^ | 43.25^***^ | 73.45 |
| TPC | 2.29^***^ | 2293.16^***^ | 69.62 |  | TGW | 4.00^***^ | 57.04^***^ | 80.00 |
| RSPC | 1.49^**^ | 117.29^***^ | 59.80 |  | GWP | 1.34^**^ | 46.23^***^ | 57.19 |
| SPutE | 1.51^**^ | 270.14^***^ | 60.08 |  | StWP | 1.68^***^ | 46.52^***^ | 54.05 |
| RPutE | 1.43^**^ | 523.11^***^ | 58.78 |  | GPC | 1.43^**^ | 189.67^***^ | 58.81 |
| TPutE | 1.67^***^ | 443.94^***^ | 62.51 |  | StPC | 1.41^***^ | 176.81^***^ | 52.61 |
|  |  |  |  |  | GPutE | 1.67^***^ | 6.23^**^ | 62.52 |
|  |  |  |  |  | StPutE | 1.46^***^ | 30.10^***^ | 51.12 |

^**^, and ^***^ indicate the significance at *p*≤0.01 and *p*≤0.001, respectively; *h_B_^2^* =σg^2^/ (σg^2^+σe^2^), σg^2^ was the genotypic variance and σe^2^ was the total error variance.
