# Supplementary material for: QTL Mapping for Phosphorus Efficiency and Morphological Traits at Seedling and Maturity Stages in Wheat
Source: Front Plant Sci. 2017 Apr 24;8:614. doi: 10.3389/fpls.2017.00614 (PMC5402226; doi:10.3389/fpls.2017.00614)
Supplement: Supplementary file 4 [file Table4.DOCX]

**Table S4** Spearman correlation coefficients (*r*) between investigated traits

**Table S4-1** Spearman correlation coefficients (*r*) between investigated traits under hydroponic culture trials

| Traits | SDW | RDW | TDW | RSDW | SPC | RPC | TPC | RSPC | SPutE | RPutE |
| --- | --- | --- | --- | --- | --- | --- | --- | --- | --- | --- |
| RDW | 0.517^**^ |  |  |  |  |  |  |  |  |  |
| TDW | 0.987^**^ | 0.640^**^ |  |  |  |  |  |  |  |  |
| RSDW | -0.615^**^ | 0.306^**^ | -0.490^**^ |  |  |  |  |  |  |  |
| SPC | 0.646^**^ | 0.297^**^ | 0.633^**^ | -0.446^**^ |  |  |  |  |  |  |
| RPC | 0.448^**^ | 0.515^**^ | 0.498^**^ | -0.032 | 0.731^**^ |  |  |  |  |  |
| TPC | 0.647^**^ | 0.332^**^ | 0.642^**^ | -0.411^**^ | 0.995^**^ | 0.785^**^ |  |  |  |  |
| RSPC | -0.384^**^ | 0.148^**^ | -0.315^**^ | 0.575^**^ | -0.510^**^ | 0.130^**^ | -0.444^**^ |  |  |  |
| SPutE | 0.199^**^ | 0.156^**^ | 0.203^**^ | -0.085^*^ | -0.549^**^ | -0.442^**^ | -0.545^**^ | 0.231^**^ |  |  |
| RPutE | -0.073^*^ | 0.276^**^ | -0.019 | 0.333^**^ | -0.596^**^ | -0.624^**^ | -0.613^**^ | 0.047 | 0.701^**^ |  |
| TPutE | 0.139^**^ | 0.203^**^ | 0.159^**^ | 0.026 | -0.591^**^ | -0.487^**^ | -0.587^**^ | 0.230^**^ | 0.985^**^ | 0.799^**^ |

^*^, and ^**^ indicate the significance at *p*≤0.05 and *p*≤0.01, respectively.

**Table S4-2** Spearman correlation coefficients (*r*) between investigated traits under field trials

| Traits | SN | PH | SL | FSS | SSS | GN | TGW | GWP | StWP | GPC | StPC | GPutE |
| --- | --- | --- | --- | --- | --- | --- | --- | --- | --- | --- | --- | --- |
| PH | -0.069 |  |  |  |  |  |  |  |  |  |  |  |
| SL | 0.146^**^ | 0.032 |  |  |  |  |  |  |  |  |  |  |
| FSS | -0.092^*^ | 0.232^**^ | 0.438^**^ |  |  |  |  |  |  |  |  |  |
| SSS | 0.068 | 0.092^*^ | -0.085^*^ | -0.105^**^ |  |  |  |  |  |  |  |  |
| GN | -0.102^**^ | -0.107^**^ | 0.487^**^ | 0.478^**^ | -0.328^**^ |  |  |  |  |  |  |  |
| TGW | -0.321^**^ | 0.502^**^ | -0.134^**^ | 0.160^**^ | 0.091^*^ | -0.100^**^ |  |  |  |  |  |  |
| GWP | 0.324^**^ | 0.252^**^ | -0.024 | 0.018 | 0.034 | -0.029 | 0.203^**^ |  |  |  |  |  |
| StWP | -0.014 | 0.449^**^ | -0.160^**^ | 0.028 | 0.063 | -0.092^*^ | 0.406^**^ | 0.458^**^ |  |  |  |  |
| GPC | 0.172^**^ | 0.190^**^ | -0.047 | -0.002 | 0.038 | -0.001 | 0.252^**^ | 0.804^**^ | 0.082^*^ |  |  |  |
| StPC | 0.099^*^ | 0.342^**^ | -0.115^**^ | 0.058 | 0.030 | -0.062 | 0.356^**^ | 0.201^**^ | 0.707^**^ | 0.302^**^ |  |  |
| GPutE | 0.323^**^ | 0.196^**^ | -0.008 | 0.046 | 0.024 | -0.082^*^ | 0.091^*^ | 0.775^**^ | 0.257^**^ | 0.297^**^ | -0.014 |  |
| StPutE | 0.113^**^ | 0.202^**^ | -0.067 | -0.027 | 0.038 | -0.028 | 0.119^**^ | 0.351^**^ | 0.499^**^ | 0.223^**^ | -0.172^**^ | 0.357^**^ |

^*^, and ^**^ indicate the significance at *p*≤0.05 and *p*≤0.01, respectively.

**Table S4-3** Spearman correlation coefficients (*r*) between seedling traits and adult traits

| Traits | SN | PH | SL | FSS | SSS | GN | TGW | GWP | StWP | GPC | StPC | GPutE | StPutE |
| --- | --- | --- | --- | --- | --- | --- | --- | --- | --- | --- | --- | --- | --- |
| SDW | 0.035 | 0.282^**^ | -0.158^**^ | 0.003 | 0.095^*^ | -0.179^**^ | 0.316^**^ | 0.231^**^ | 0.243^**^ | 0.296^**^ | 0.240^**^ | 0.078^*^ | 0.023 |
| RDW | -0.246^**^ | 0.242^**^ | -0.035 | 0.159^**^ | 0.048 | -0.062 | 0.345^**^ | -0.023 | 0.130^**^ | -0.014 | 0.107^**^ | -0.042 | 0.034 |
| TDW | -0.014 | 0.300^**^ | -0.151^**^ | 0.034 | 0.091 | -0.174^**^ | 0.348^**^ | 0.203^**^ | 0.241^**^ | 0.265^**^ | 0.232^**^ | 0.064 | 0.029 |
| RSDW | -0.252^**^ | -0.092^*^ | 0.130^**^ | 0.139^**^ | -0.058 | 0.143^**^ | -0.043 | -0.258^**^ | -0.158^**^ | -0.327^**^ | -0.178^**^ | -0.103^**^ | 0.003 |
| SPC | -0.136^**^ | 0.403^**^ | -0.215^**^ | 0.069 | 0.054 | -0.106^**^ | 0.479^**^ | 0.228^**^ | 0.446^**^ | 0.385^**^ | 0.577^**^ | -0.019 | -0.084^*^ |
| RPC | -0.220^**^ | 0.373^**^ | -0.122^**^ | 0.156^**^ | 0.087^*^ | -0.059 | 0.454^**^ | 0.098^**^ | 0.337^**^ | 0.218^**^ | 0.438^**^ | -0.051 | -0.067 |
| TPC | -0.149^**^ | 0.415^**^ | -0.211^**^ | 0.081^*^ | 0.060 | -0.103^**^ | 0.494^**^ | 0.227^**^ | 0.450^**^ | 0.372^**^ | 0.575^**^ | -0.015 | -0.076^*^ |
| RSPC | -0.035 | -0.115^**^ | 0.175^**^ | 0.118^**^ | 0.041 | 0.083^*^ | -0.146^**^ | -0.159^**^ | -0.220^**^ | -0.229^**^ | -0.283^**^ | -0.018 | 0.045 |
| SPutE | 0.216^**^ | -0.201^**^ | 0.109^**^ | -0.071 | 0.025 | -0.053 | -0.273^**^ | -0.014 | -0.303^**^ | -0.155^**^ | -0.499^**^ | 0.134^*^ | 0.162^**^ |
| RPutE | 0.038 | -0.215^**^ | 0.103^**^ | -0.022 | -0.065 | -0.001 | -0.217^**^ | -0.140^**^ | -0.282^**^ | -0.283^**^ | -0.453^**^ | 0.038 | 0.133^**^ |
| TPutE | 0.180^**^ | -0.213^**^ | 0.114^**^ | -0.051 | 0.006 | -0.043 | -0.271^**^ | -0.048 | -0.317^**^ | -0.205^**^ | -0.516^**^ | 0.122^**^ | 0.162^**^ |

^*^, and ^**^ indicate the significance at *p*≤0.05 and *p*≤0.01, respectively.

**Table S4-4** Spearman correlation coefficients (*r*) between relative traits, and between relative and PE traits under LP treatment of field trials

| Traits | Rsn | Rph | Rgn | Rtgw | Rgwp | Rstwp |
| --- | --- | --- | --- | --- | --- | --- |
| Rph | 0.310^**^ |  |  |  |  |  |
| Rgn | -0.104 | 0.203^**^ |  |  |  |  |
| Rtgw | 0.038 | 0.045 | 0.077 |  |  |  |
| Rgwp | 0.150^*^ | 0.024 | 0.013 | 0.081 |  |  |
| Rstwp | 0.193^**^ | 0.212^**^ | 0.050 | 0.040 | 0.377^**^ |  |
| GPC | -0.109 | -0.106 | 0.008 | -0.096 | 0.581^**^ | 0.173^*^ |
| StPC | 0.020 | 0.012 | -0.077 | 0.043 | 0.002 | 0.372^**^ |
| GPutE | 0.059 | 0.016 | -0.033 | 0.194^**^ | 0.563^**^ | 0.178^*^ |
| StPutE | 0.151^*^ | 0.229^**^ | 0.076 | -0.024 | 0.266^**^ | 0.489^**^ |

^*^, and ^**^ indicate the significance at *p*≤0.05 and *p*≤0.01, respectively.
