# Supplementary material for: QTL Mapping for Phosphorus Efficiency and Morphological Traits at Seedling and Maturity Stages in Wheat
Source: Front Plant Sci. 2017 Apr 24;8:614. doi: 10.3389/fpls.2017.00614 (PMC5402226; doi:10.3389/fpls.2017.00614)
Supplement: Supplementary file 5 [file Table5.DOCX]

**Table S5** Additive QTLs for seedling, maturity and relative traits detected in different P treatments.

| Traits | QTLs | Treatments | Marker intervals ^a^ | | LODs | Additive effects ^b^ | *R^2^* (%) |
| --- | --- | --- | --- | --- | --- | --- | --- |
| ***Seedling traits*** | | | | | | | |
| RDW | *QRdw-1B* | LPAV | *D-3952804-D-1382734* | | 3.92 | -0.57 | 6.40 |
|  | *QRdw-3B.1* | LP2 | *D-3950571-D-1008756* | | 4.57 | 0.86 | 9.56 |
|  | *QRdw-3B.2* | LPAV | *D-1095838-S-3023002* | | 4.18 | 0.63 | 8.47 |
|  | *QRdw-3D* | NP1 | *S-3024408-D-3534001* | | 5.17 | 0.76 | 10.21 |
|  | *QRdw-4B* | NPAV | *D-1084202-wmc657* | | 4.14 | 0.54 | 6.78 |
|  | *QRdw-5D.1* | LP1 | *D-1116418-D-2323329* | | 4.57 | -0.63 | 7.91 |
|  |  | LPAV | *D-1116418-D-2323329* | | 5.82 | -0.68 | 10.12 |
|  |  | LP2 | *D-2323329-D-3948435* | | 5.39 | -0.91 | 10.48 |
|  |  | NP1 | *D-2323329-D-3948435* | | 9.46 | -0.92 | 15.23 |
|  | *QRdw-5D.2* | NP2 | *D-1108150-D-3025275* | | 4.15 | -0.64 | 7.57 |
|  |  | NPAV | *S-1062048-D-1116488* | | 8.40 | -0.79 | 14.20 |
|  | *QRdw-6B* | NP1 | *D-3953053-D-991702* | | 7.48 | -0.82 | 11.59 |
| SDW | *QSdw-4B.1* | NPAV | *D-1051883-D-1113185* | | 4.53 | -3.45 | 7.80 |
|  | *QSdw-4B.2* | NP1 | *S-3024027-S-1078626* | | 11.93 | -6.40 | 19.10 |
|  |  | LP1 | *S-1040960-D-1083795* | | 7.44 | -5.05 | 14.06 |
|  |  | NP2 | *D-3940950-S-3024027* | | 4.60 | -4.18 | 8.71 |
|  |  | NPAV | *S-3024027-S-1078626* | | 9.46 | -4.97 | 15.66 |
|  |  | LPAV | *D-3940950-S-3024027* | | 7.80 | -3.78 | 13.67 |
|  | *QSdw-4B.3* | LP2 | *D-4008856-D-1138250* | | 4.26 | -2.87 | 8.50 |
|  | *QSdw-5D.1* | NPAV | *D-1055236-D-3956782* | | 4.95 | -3.40 | 7.87 |
|  |  | LPAV | *D-3956782-D-1117581* | | 4.53 | -2.67 | 7.63 |
|  | *QSdw-5D.2* | NP1 | *D-1261108-S-1163901* | | 6.13 | -4.33 | 9.35 |
|  | *QSdw-6A.1* | LPAV | *D-1081976-S-1149480* | | 5.75 | -2.98 | 9.70 |
|  | *QSdw-6A.2* | NP1 | *D-3937732-S-3033958* | | 4.51 | -3.66 | 6.67 |
|  | *QSdw-6B.1* | LP2 | *D-4329798-D-3960242* | | 4.16 | -2.72 | 8.04 |
|  | *QSdw-6B.2* | NP1 | *D-3953053-D-991702* | | 7.18 | -4.66 | 10.82 |
|  |  | NPAV | *D-3953053-D-991702* | | 5.90 | -3.77 | 9.48 |
| RSDW | *QRsdw-1A* | LP2 | *Excalibur_c47013_1503-* | | 4.07 | 0.01 | 4.52 |
|  |  |  | *wsnp_Ex_rep_c102067_87314043* | | |  |  |
|  | *QRsdw-3B* | LP2 | *D-1106251-D-1122986* | 5.30 | | 0.01 | 6.10 |
|  |  | LPAV | *D-1106251-D-1122986* | 6.01 | | 0.01 | 6.40 |
|  | *QRsdw-4B* | NP1 | *D-3940950-S-3024027* | 31.55 | | 0.04 | 50.28 |
|  |  | LP1 | *D-3940950-S-3024027* | 28.81 | | 0.04 | 42.84 |
|  |  | NP2 | *D-3940950-S-3024027* | 29.47 | | 0.02 | 44.21 |
|  |  | LP2 | *D-3940950-S-3024027* | 16.81 | | 0.02 | 26.91 |
|  |  | NPAV | *D-3940950-S-3024027* | 36.00 | | 0.03 | 51.68 |
|  |  | LPAV | *D-3940950-S-3024027* | 32.86 | | 0.03 | 46.84 |
|  | *QRsdw-7B* | NP2 | *D-1105223-D-1107283* | 4.68 | | -0.01 | 5.27 |
| TDW | *QTdw-1A* | NP2 | *wPt-734027-D-3934878* | 4.54 | | 5.03 | 8.75 |
|  | *QTdw-4B* | NP1 | *S-3024027-S-1078626* | 7.25 | | -5.64 | 11.76 |
|  |  | NPAV | *S-3024027-S-1078626* | 6.27 | | -4.49 | 10.36 |
|  | *QTdw-5D* | NP1 | *D-2323329-D-3948435* | 6.31 | | -5.03 | 10.27 |
|  |  | NPAV | *D-1055236-D-3956782* | 5.45 | | -4.07 | 9.06 |
|  | *QTdw-6A.1* | NP1 | *D-1118135-S-1006219* | 6.34 | | -5.03 | 10.17 |
|  |  | LP1 | *S-1006219-S-1079131* | 7.21 | | -10.48 | 14.75 |
|  |  | LPAV | *D-1081976-S-1149480* | 6.20 | | -3.71 | 11.34 |
|  | *QTdw-6A.2* | NP1 | *S-1126245-D-1252783* | 4.14 | | -3.94 | 6.46 |

^a^ Marker interval is the interval of LOD peak value for QTLs; ^b^ Positive additive effect is increasing effect contributed by TN18, negative additive effect is increasing effect contributed by LM6.

**Table S5** Continued-1

| Traits | QTLs | Treatments | Marker intervals ^a^ | LODs | Additive effects ^b^ | *R^2^* (%) |
| --- | --- | --- | --- | --- | --- | --- |
| ***Seedling traits*** | | | | | | |
|  | *QTdw-6B.1* | LP2 | *D-4329798-D-3960242* | 4.54 | -3.52 | 8.91 |
|  | *QTdw-6B.2* | NPAV | *D-3953053-D-991702* | 6.24 | -4.44 | 10.48 |
| RPC | *QRpc-2D* | LPAV | *D-3533182-cfd62* | 4.84 | 0.01 | 9.06 |
|  | *QRpc-3B* | NP1 | *S-3064384-S-1007253* | 5.75 | 0.01 | 10.80 |
|  | *QRpc-4B.1* | NP2 | *D-3953244-D-4002948* | 4.06 | 0.01 | 7.67 |
|  | *QRpc-4B.2* | NPAV | *D-3022151-S-1040960* | 6.18 | 0.01 | 14.18 |
|  | *QRpc-5D.1* | LPAV | *S-1081384-S-3028008* | 6.24 | -0.01 | 11.66 |
|  |  | LP1 | *S-3028008-S-1055033* | 4.02 | -0.01 | 8.45 |
|  | *QRpc-5D.2* | NP1 | *D-3533903-D-1261108* | 4.26 | -0.01 | 7.96 |
|  | *QRpc-7A* | LPAV | *wsnp_Ku_c26530_36497050-D-1098404* | 4.07 | 0.00 | 7.47 |
|  | *QRpc-7B* | LP2 | *D-3947300-D-3936306* | 4.09 | 0.02 | 10.01 |
| SPC | *QSpc-1A* | LPAV | *wPt-667566-swes139* | 3.88 | -0.02 | 7.77 |
|  | *QSpc-1D.1* | LP1 | *D-2251236-D-3957613* | 5.74 | -0.04 | 11.30 |
|  | *QSpc-1D.2* | LP1 | *D-1218473-D-2294989* | 4.27 | 0.03 | 8.09 |
|  | *QSpc-5D* | NP1 | *D-1055236-D-3956782* | 4.51 | -0.04 | 8.22 |
|  | *QSpc-6A* | NPAV | *S-1378596-D-3952397* | 6.92 | -0.05 | 14.05 |
|  | *QSpc-6B* | NP1 | *D-3953053-D-991702* | 6.26 | -0.05 | 11.52 |
|  |  | LPAV | *D-3384907-D-1393612* | 4.13 | -0.03 | 12.48 |
|  | *QSpc-7B* | NPAV | *D-1304087-D-1113800* | 5.65 | 0.07 | 18.27 |
| RSPC | *QRspc-1D* | LP1 | *D-3959209-D-2251236* | 5.04 | 0.05 | 10.13 |
|  | *QRspc-4A* | LPAV | *D-3934345-D-2276787* | 4.37 | -0.03 | 8.38 |
|  | *QRspc-4B.1* | NP2 | *D-3022151-S-1040960* | 4.21 | 0.01 | 9.69 |
|  | *QRspc-4B.2* | NP1 | *D-4008856-D-1138250* | 7.64 | 0.03 | 14.19 |
|  |  | NPAV | *S-3024027-S-1078626* | 9.87 | 0.02 | 18.25 |
|  | *QRspc-6A* | LP2 | *S-3026216-D-1722489* | 3.90 | -0.02 | 8.46 |
|  | *QRspc-6B* | LP1 | *D-2265719-D-1263293* | 4.15 | -0.04 | 7.76 |
|  | *QRspc-7B* | NP2 | *D-3950701-D-1126507* | 5.60 | -0.01 | 10.74 |
| TPC | *QTpc-1A* | LPAV | *wPt-667566-swes139* | 3.85 | -0.02 | 7.42 |
|  | *QTpc-1D* | LP1 | *D-1242418-D-3944973* | 4.18 | -0.03 | 9.02 |
|  | *QTpc-6A* | NPAV | *S-1378596-D-3952397* | 4.61 | -0.05 | 9.37 |
|  | *QTpc-6B.1* | LP2 | *D-1106806-D-3384656* | 4.70 | -0.04 | 9.82 |
|  | *QTpc-6B.2* | NP1 | *D-3953053-D-991702* | 7.47 | -0.06 | 13.86 |
|  |  | LPAV | *D-3384907-D-1393612* | 5.05 | -0.03 | 14.54 |
| RPutE | *QRpute-1A.1* | LP2 | *wPt-667566-swes139* | 5.22 | -0.27 | 11.30 |
|  | *QRpute-1A.2* | LP2 | *wPt-5160-S-985876* | 5.08 | -0.31 | 14.55 |
|  | *QRpute-4A* | NP2 | *D-1410594-D-1166619* | 4.11 | -0.22 | 8.29 |
|  | *QRpute-6B* | LP1 | *D-1095545-S-1006033* | 6.46 | -0.85 | 12.80 |
|  |  | LPAV | *D-1095545-S-1006033* | 6.42 | -0.47 | 12.82 |
| SPutE | *QSpute-1D* | LP2 | *D-3959209-D-2251236* | 4.67 | 3.94 | 10.24 |
|  |  | LPAV | *D-3959209-D-2251236* | 4.35 | 1.96 | 9.07 |
|  | *QSpute-4B.1* | LP2 | *D-1212306-D-1003776* | 3.94 | 0.49 | 6.98 |
|  | *QSpute-4B.2* | NP1 | *S-3024027-S-1078626* | 16.66 | -1.16 | 30.31 |
|  |  | LP1 | *D-3940950-S-3024027* | 9.79 | -0.83 | 18.75 |
|  |  | NP2 | *D-4008856-D-1138250* | 14.96 | -0.76 | 26.22 |
|  |  | NPAV | *D-3940950-S-3024027* | 18.95 | -0.95 | 32.84 |
|  | *QSpute-4D* | NP1 | *S-1297707-Kukri_c444_833* | 4.11 | -0.55 | 7.25 |

**Table S5** Continued-2

| Traits | QTLs | Treatments | Marker intervals ^a^ | LODs | Additive effects ^b^ | *R^2^* (%) |
| --- | --- | --- | --- | --- | --- | --- |
| ***Seedling traits*** | | | | | | |
|  | *QSpute-5D* | LP2 | *S-1055033-D-4329475* | 4.64 | -0.54 | 8.36 |
|  | *QSpute-6B* | LP1 | *S-2261574-D-4329433* | 4.84 | -3.88 | 11.33 |
|  |  | LPAV | *D-4329433-D-1217508* | 4.51 | -1.94 | 10.12 |
| TPutE | *QTpute-1D* | LP1 | *D-3959209-D-2251236* | 4.10 | 3.53 | 8.71 |
|  | *QTpute-3B* | NP2 | *D-1095838-S-3023002* | 4.05 | 0.45 | 7.68 |
|  | *QTpute-4B.1* | NP1 | *D-1051883-D-1113185* | 5.58 | -0.74 | 10.51 |
|  |  | NPAV | *D-1051883-D-1113185* | 5.32 | -0.57 | 10.13 |
|  |  | NP2 | *D-1113185-Ku_c63300_1309* | 4.29 | -0.54 | 10.49 |
|  | *QTpute-4B.2* | NP1 | *S-3024027-S-1078626* | 10.94 | -1.02 | 19.58 |
|  |  | NP2 | *D-4008856-D-1138250* | 13.00 | -0.82 | 23.09 |
|  |  | LP2 | *S-3024027-S-1078626* | 6.11 | -0.67 | 11.33 |
|  |  | NPAV | *S-3024027-S-1078626* | 15.07 | -0.95 | 26.23 |
|  | *QTpute-4B.3* | NP2 | *D-1302339-D-1375793* | 4.47 | -0.51 | 9.08 |
|  | *QTpute-5D* | NP1 | *D-2323329-D-3948435* | 4.28 | -0.58 | 7.19 |
|  |  | NPAV | *D-1695705-D-1233019* | 4.37 | -0.45 | 6.76 |
|  | *QTpute-6B* | LPAV | *D-4329433-D-1217508* | 4.93 | -2.00 | 11.29 |
| ***Mature traits*** | | | | | | |
| PH | *QPh-1D* | LP3 | *D-1107521-D-1254464* | 4.23 | 1.16 | 9.06 |
|  | *QPh-4B.2* | NP4 | *D-1673295-S-3941408* | 9.59 | -2.13 | 15.27 |
|  |  | LPAV | *D-1673295-S-3941408* | 5.77 | -1.45 | 10.08 |
|  | *QPh-5A* | NP3 | *S-1220783-S-1088638* | 6.39 | -1.36 | 12.75 |
|  | *QPh-5B* | NPAV | *S-989092-D-984417* | 8.75 | -1.58 | 15.77 |
|  | *QPh-6B* | LP3 | *D-1102453-D-3033837* | 4.89 | 1.24 | 10.89 |
|  | *QPh-6D* | NP4 | *D-1246541-D-1132926* | 7.74 | 1.86 | 12.03 |
|  |  | LP4 | *D-1129747-D-2265140* | 7.49 | 2.07 | 13.49 |
|  |  | LPAV | *D-1129747-D-2265140* | 5.10 | 1.19 | 8.75 |
| SL | *QSl-2D* | NPAV | *D-1214437-D-1103717* | 4.34 | -0.15 | 8.02 |
|  |  | NP4 | *D-1071500-D-3953113* | 5.93 | -0.22 | 11.93 |
|  | *QSl-4A.1* | LP4 | *wPt-731639-D-4329307* | 4.25 | 0.18 | 7.53 |
|  | *QSl-4A.2* | NPAV | *S-3943887-swes620a* | 4.59 | 0.15 | 8.40 |
|  | *QSl-4B* | LPAV | *S-1040960-D-1083795* | 4.93 | 0.16 | 8.39 |
|  | *QSl-5A* | NP4 | *D-1216984-D-1361259* | 4.10 | -0.18 | 8.56 |
|  | *QSl-5B* | NP4 | *Excalibur_rep_c67473_320-D-3934137* | 3.93 | 0.18 | 7.71 |
|  | *QSl-7A* | LPAV | *wPt-4617-wmc388* | 5.43 | 0.18 | 10.68 |
|  | *QSl-7D.1* | LPAV | *cfd14-D-3533458* | 6.69 | 0.19 | 11.56 |
|  | *QSl-7D.2* | LP3 | *wPt-7508-D-3956292* | 4.00 | 0.18 | 7.49 |
|  |  | NP3 | *wPt-7508-D-3956292* | 4.52 | 0.19 | 8.29 |
|  | *QSl-7D.3* | LPAV | *D-4329569-S-989952* | 4.45 | 0.16 | 8.41 |
|  | *QSl-7D.4* | LP3 | *S-1101488-D-2323106* | 4.67 | 0.21 | 10.30 |
| SN | *QSn-2A* | LP4 | *D-3026757-D-3961082* | 4.23 | -0.76 | 8.52 |
|  | *QSn-4A* | LPAV | *wsnp_Ra_c16476_25132652-wPt-0162* | 6.79 | -1.02 | 15.57 |
|  | *QSn-4B.1* | LP3 | *D-1108965-D-3024350* | 5.36 | -0.84 | 10.35 |
|  | *QSn-4B.2* | NP3 | *D-3022151-S-1040960* | 4.26 | -0.95 | 11.12 |
|  |  | NPAV | *D-3022151-S-1040960* | 13.50 | -1.27 | 30.08 |
|  | *QSn-4B.3* | NP4 | *S-1078626-D-1673295* | 11.04 | -1.26 | 19.20 |
|  | *QSn-5A.1* | NP3 | *D-1207347-S-1087661* | 5.57 | 1.18 | 14.65 |
|  |  | LP3 | *S-1087661-D-1089337* | 6.10 | 0.92 | 12.06 |

**Table S5** Continued-3

| Traits | QTLs | Treatments | Marker intervals ^a^ | LODs | Additive effects ^b^ | *R^2^* (%) |
| --- | --- | --- | --- | --- | --- | --- |
| ***Mature traits*** | | | | | | |
|  | *QSn-5A.2* | NP4 | *D-1092382-wsnp_RFL_Contig4307_5006558* | 5.41 | 0.84 | 9.08 |
| GN | *QGn-1D* | NP4 | *wmc429-D-1238586* | 4.50 | -1.68 | 7.79 |
|  |  | NPAV | *wmc429-D-1238586* | 4.38 | -1.14 | 7.15 |
|  | *QGn-2A.1* | NP3 | *S-988596-D-3950218* | 4.55 | -1.55 | 8.96 |
|  | *QGn-2A.2* | LP3 | *S-2253907-D-3024109* | 3.81 | -2.11 | 8.11 |
|  | *QGn-4A* | NP4 | *Excalibur_c8658_335-Ex_c101416_378* | 5.10 | 1.74 | 8.83 |
|  |  | NPAV | *S-1233192-swes1060* | 5.02 | 1.30 | 9.43 |
|  | *QGn-4B.1* | LP4 | *S-1040960-D-1083795* | 8.00 | 2.41 | 14.89 |
|  | *QGn-4B.2* | NP4 | *S-3941408-D-4008856* | 4.09 | 1.68 | 7.08 |
|  |  | NPAV | *S-3941408-D-4008856* | 10.28 | 1.88 | 18.77 |
|  | *QGn-7B* | NP3 | *D-1390136-S-991542* | 7.00 | 1.82 | 13.06 |
|  |  | LP3 | *D-1390136-S-991542* | 4.91 | 2.39 | 10.36 |
| FSS | *QFss-2A* | LPAV | *D-1151532-D-1163690* | 4.19 | -0.21 | 7.62 |
|  | *QFss-3B* | LP4 | *D-2275562-BobWhite_c39144_347* | 5.83 | 0.34 | 10.80 |
|  | *QFss-4A* | NP4 | *IAAV3080-Kukri_c19562_127* | 3.23 | 0.30 | 6.73 |
|  | *QFss-5B* | NPAV | *D-1092353-S-3030622* | 4.78 | 0.37 | 8.91 |
|  | *QFss-7D.1* | NP3 | *Excalibur_c55782_55-swes1089* | 5.53 | 0.28 | 10.68 |
|  | *QFss-7D.2* | NPAV | *wPt-7508-D-3956292* | 3.50 | 0.22 | 7.56 |
|  | *QFss-7D.3* | NP4 | *S-3022993-D-4329404* | 4.20 | 0.35 | 8.84 |
|  | *QFss-7D.4* | LP4 | *S-989952-D-3533947* | 4.94 | 0.31 | 8.93 |
|  |  | LPAV | *S-1286843-D-1247149* | 5.18 | 0.23 | 9.52 |
| SSS | *QSss-1A.1* | NP4 | *D-1090522-swes1058* | 4.67 | -0.08 | 8.59 |
|  | *QSss-1A.2* | NPAV | *BS00064638_51-S-1224205* | 4.53 | -0.06 | 8.43 |
|  | *QSss-1A.3* | NP4 | *S-1206867-D-1218238* | 5.91 | -0.09 | 11.36 |
|  |  | NPAV | *S-1206867-D-1218238* | 6.40 | -0.07 | 11.52 |
|  | *QSss-1B* | LPAV | *D-3938786-D-2280342* | 4.66 | -0.06 | 9.35 |
|  | *QSss-4B* | LP3 | *D-1139998-D-3955800* | 6.09 | 0.09 | 11.46 |
|  | *QSss-5D* | LPAV | *S-3028008-S-1055033* | 4.23 | -0.05 | 8.30 |
|  | *QSss-6B.1* | NP3 | *Tdurum_contig33428_272-* | 6.42 | -0.10 | 13.69 |
|  |  |  | *BS00090073_51* |  |  |  |
|  | *QSss-6B.2* | NP3 | *Excalibur_rep_c102994_1003-wPt-2175* | 3.90 | 0.08 | 7.54 |
|  | *QSss-7B* | LP3 | *wPt-4045-Kukri_c10430_642* | 5.39 | 0.08 | 9.90 |
| TGW | *QTgw-1B* | LPAV | *S-2261197-D-992533* | 6.88 | -1.16 | 12.43 |
|  | *QTgw-1D.1* | NP4 | *D-1091928-D-1084615* | 5.10 | 1.17 | 9.13 |
|  | *QTgw-1D.2* | LP4 | *BS00073257_51-D-3960969* | 7.08 | 1.44 | 14.21 |
|  | *QTgw-2A* | NPAV | *D-3957579-S-1248551* | 4.12 | -0.78 | 7.50 |
|  | *QTgw-2D.1* | LPAV | *cfd233-D-1125868* | 6.13 | 1.01 | 10.64 |
|  |  | LP4 | *cfd233-D-1125868* | 4.99 | 1.13 | 9.21 |
|  | *QTgw-2D.2* | LP4 | *D-1088275-S-1054524* | 5.62 | 1.20 | 9.79 |
|  | *QTgw-4B.1* | LP4 | *wPt-744595-wPt-7233* | 4.50 | 1.02 | 7.39 |
|  | *QTgw-4B.2* | NP3 | *D-3570086-D-1098436* | 4.89 | 1.14 | 9.78 |
|  | *QTgw-5B* | NPAV | *D-1104538-D-3384695* | 4.65 | -0.83 | 8.64 |
|  | *QTgw-5D* | LP4 | *D-3960760-D-1229814* | 4.07 | -0.98 | 6.70 |

**Table S5** Continued-4

| Traits | | QTLs | | Treatments | Marker intervals ^a^ | LODs | Additive effects ^b^ | *R^2^* (%) |
| --- | --- | --- | --- | --- | --- | --- | --- | --- |
| ***Mature traits*** | | | | | | | | |
|  | *QTgw-6A* | | LP4 | | *D-1256179-D-1118135* | 3.84 | -0.95 | 6.41 |
|  | *QTgw-6B* | | NP4 | | *S-1094839-wPt-9784* | 4.60 | -1.12 | 8.44 |
|  | *QTgw-7A* | | LPAV | | *D-1071990-D-1220488* | 3.89 | -0.80 | 6.67 |
| GWP | *QGwp-2A* | | LP3 | | *S-1074649-D-3940454* | 6.79 | -2.28 | 14.82 |
|  | *QGwp-3A* | | NP4 | | *D-4004662-D-3533086* | 5.57 | -2.43 | 9.83 |
|  | *QGwp-4B* | | NPAV | | *D-3022151-S-1040960* | 5.24 | -1.42 | 11.34 |
|  |  | | NP4 | | *D-3940950-S-3024027* | 6.15 | -2.08 | 10.83 |
|  | *QGwp-5A.1* | | NP4 | | *D-1266712-D-1215862* | 8.09 | 2.45 | 15.37 |
|  |  | | NPAV | | *D-1266712-D-1215862* | 7.07 | 1.49 | 12.62 |
|  | *QGwp-5A.2* | | NP4 | | *wsnp_Ex_c2689_4986791-S-2261145* | 5.71 | 2.28 | 13.30 |
| StWP | *QStwp-5B.1* | | LP4 | | *S-2282007-S-2255854* | 4.63 | 4.65 | 10.25 |
|  | *QStwp-5B.2* | | NP4 | | *D-2304690-D-1101787* | 4.84 | -3.29 | 10.58 |
|  | *QStwp-7A* | | LP4 | | *D-1266662-D-1116886* | 3.77 | 3.11 | 8.27 |
| GPC | *QGpc-1B* | | NPAV | | *S-1064916-D-4329409* | 5.33 | -7.51 | 9.78 |
|  | *QGpc-5A* | | NP4 | | *D-3946795-wsnp_Ku_c14275_22535693* | 7.82 | 10.16 | 15.45 |
|  |  | | NPAV | | *D-3946795-wsnp_Ku_c14275_22535693* | 5.25 | 7.52 | 10.19 |
|  | *QGpc-6B* | | NP4 | | *D-3532763-wPt-6594* | 4.28 | -7.38 | 7.94 |
|  | *QGpc-7B* | | LP3 | | *S-3029945-D-1164191* | 6.34 | 12.83 | 13.85 |
|  |  | | LPAV | | *S-3029945-D-1164191* | 4.65 | 7.91 | 9.99 |
| StPC | *QStpc-4B* | | NP4 | | *S-2275640-S-1101888* | 4.31 | 5.07 | 8.21 |
| GPutE | *QGpute-3B* | | NP3 | | *D-1254445-D-1269428* | 4.72 | -0.66 | 12.19 |
|  | *QGpute-4B.1* | | NP4 | | *D-1051883-D-1113185* | 5.46 | -0.72 | 10.90 |
|  |  | | NPAV | | *D-1051883-D-1113185* | 4.63 | -0.48 | 8.82 |
|  | *QGpute-4B.2* | | NP4 | | *S-1040960-D-1083795* | 12.02 | -1.01 | 23.54 |
|  |  | | NPAV | | *D-3940950-S-3024027* | 6.82 | -0.57 | 12.72 |
|  | *QGpute-5A.1* | | NPAV | | *D-1266712-D-1215862* | 4.46 | 0.44 | 8.04 |
|  | *QGpute-5A.2* | | NP4 | | *D-1103819-S-3022014* | 5.35 | 0.54 | 8.78 |
|  | *QGpute-5B* | | LPAV | | *wPt-4936-BS00009607_51* | 3.75 | -0.58 | 7.23 |
|  | *QGpute-7A* | | LP4 | | *D-1036713-wPt-3523* | 4.64 | 0.82 | 10.28 |
|  | *QGpute-7B* | | LP4 | | *Tdurum_contig8719_442-D-1128482* | 4.20 | 0.81 | 9.05 |
| StPutE | *QStpute-1A* | | NP4 | | *Tdurum_contig9811_127-* | 4.48 | 6.06 | 10.31 |
|  |  | |  | | *BS00063847_51* |  |  |  |
|  | *QStpute-3B.1* | | NPAV | | *D-1208515-D-1378985* | 4.96 | 4.45 | 9.48 |
|  | *QStpute-3B.2* | | NPAV | | *wPt-3921-wPt-3094* | 6.97 | -5.27 | 13.52 |
|  | *QStpute-5A* | | LP3 | | *D-1402738-D-1287504* | 3.81 | -6.33 | 8.95 |
|  | *QStpute-5B* | | NP4 | | *D-1100643-S-1047582* | 6.61 | -5.83 | 12.79 |
|  | *QStpute-6A* | | NPAV | | *S-1079131-D-1201591* | 5.17 | -3.30 | 9.49 |
|  | *QStpute-6B* | | NP4 | | *D-1095545-S-1006033* | 4.21 | -4.58 | 7.74 |
|  | *QStpute-7A* | | LP4 | | *D-1266662-D-1116886* | 4.91 | 7.39 | 10.94 |
|  | *QStpute-7D* | | NP3 | | *D-4329796-D-2244086* | 4.46 | 3.24 | 8.80 |
| ***Relative traits*** | | |  | |  |  |  |  |
| Rph | *QRph-5D* | | Trial4 | | *D-2302643-S-3024384* | 4.88 | 0.03 | 9.84 |
| Rsn | *QRsn-2B* | | Trial3 | | *wPt-2135-wPt-4368* | 5.18 | -0.07 | 10.20 |
|  | *QRsn-3A* | | Trial3 | | *wPt-1111-D-3959215* | 4.20 | 0.07 | 9.95 |
|  | *QRsn-7A* | | Trial3 | | *wPt-7122-wPt-4489* | 3.74 | 0.06 | 7.39 |
|  | *QRsn-3B.1* | | AV | | *S-1089285-S-1090569* | 3.60 | -0.11 | 7.22 |
|  | *QRsn-3B.2* | | AV | | *wPt-7961-wPt-3921* | 5.71 | -0.23 | 13.72 |
|  | *QRsn-3B.3* | | Trial4 | | *D-1318870-D-3024710* | 4.04 | 0.18 | 7.71 |
|  |  | |  | |  |  |  |  |

**Table S5** Continued-5

| Traits | QTLs | Treatments | Marker intervals ^a^ | LODs | Additive effects ^b^ | *R^2^* (%) |
| --- | --- | --- | --- | --- | --- | --- |
|  | *QRsn-3D* | AV | *D_contig68177_584-Kukri_c14642_917* | 5.63 | 0.26 | 11.49 |
|  | *QRsn-7D* | Trial4 | *Excalibur_c4508_1959-* *BS00083421_51* | 3.53 | 0.18 | 6.71 |
|  | *QRsn-6A* | AV | *D-3952397-D-1109442* | 3.49 | 0.09 | 6.84 |
| Rgwp | *QRgwp-3B* | Trial4 | *wPt-741465-BS00001400_51* | 4.41 | 0.13 | 10.29 |
|  | *QRgwp-7D* | Trial4 | *wPt-7842-wPt-731810* | 4.58 | -0.13 | 9.10 |
| Rstwp | *QRstwp-2A* | Trial4 | *D-1395795-S-988596* | 5.48 | -0.14 | 13.93 |
|  | *QRstwp-5B* | Trial4 | *Excalibur_c6967_1233-S-2282007* | 4.90 | 0.16 | 12.59 |
|  | *QRstwp-7D* | AV | *D-3023734-wPt-7368* | 3.94 | 0.14 | 11.92 |
