# Supplementary material for: QTL Mapping for Phosphorus Efficiency and Morphological Traits at Seedling and Maturity Stages in Wheat
Source: Front Plant Sci. 2017 Apr 24;8:614. doi: 10.3389/fpls.2017.00614 (PMC5402226; doi:10.3389/fpls.2017.00614)
Supplement: Supplementary file 6 [file Image1.PDF]

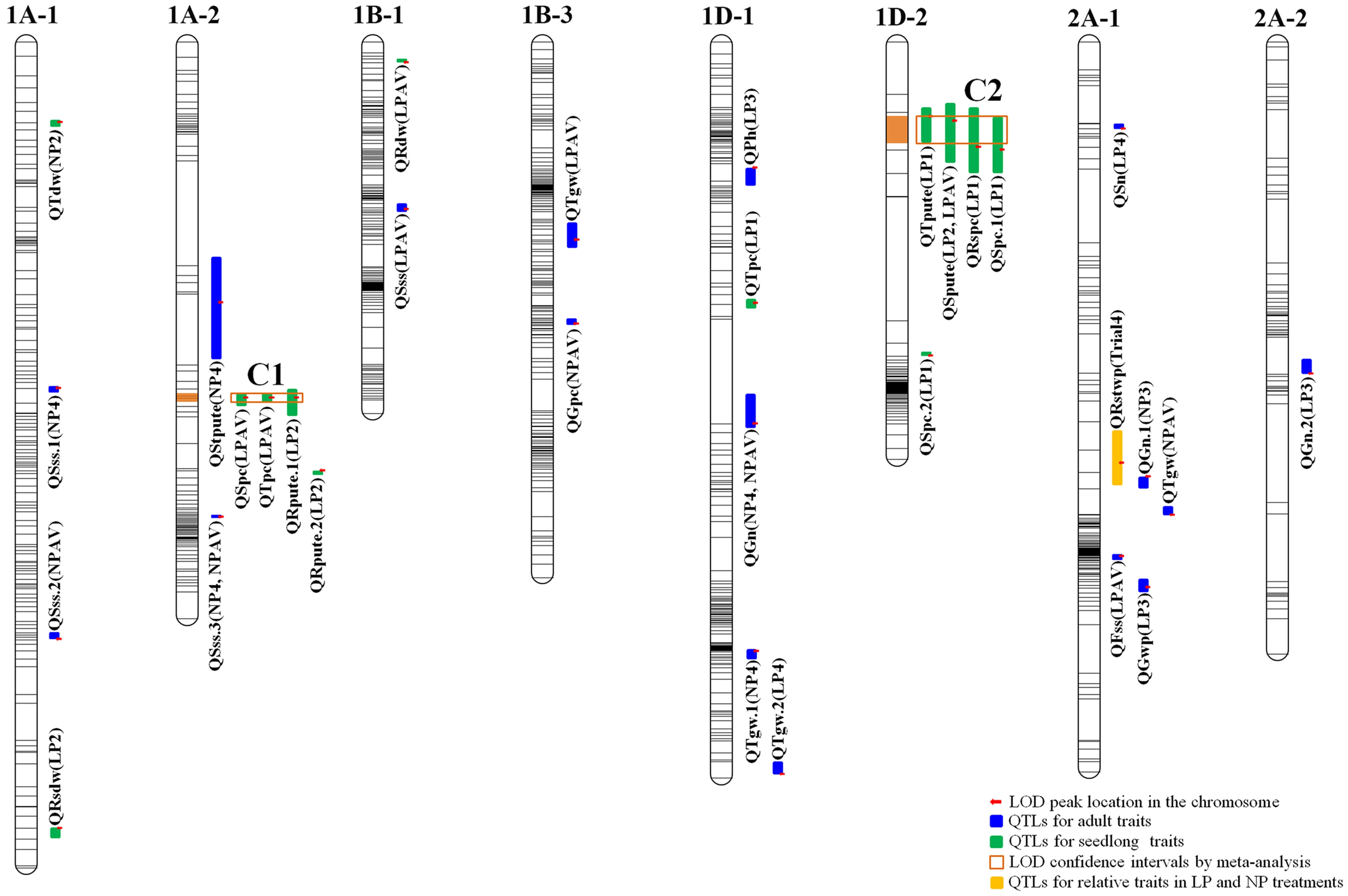

Fig. S1 Genetic linkage map and QTL locations based on RILs derived from TN 18 × LM 6.

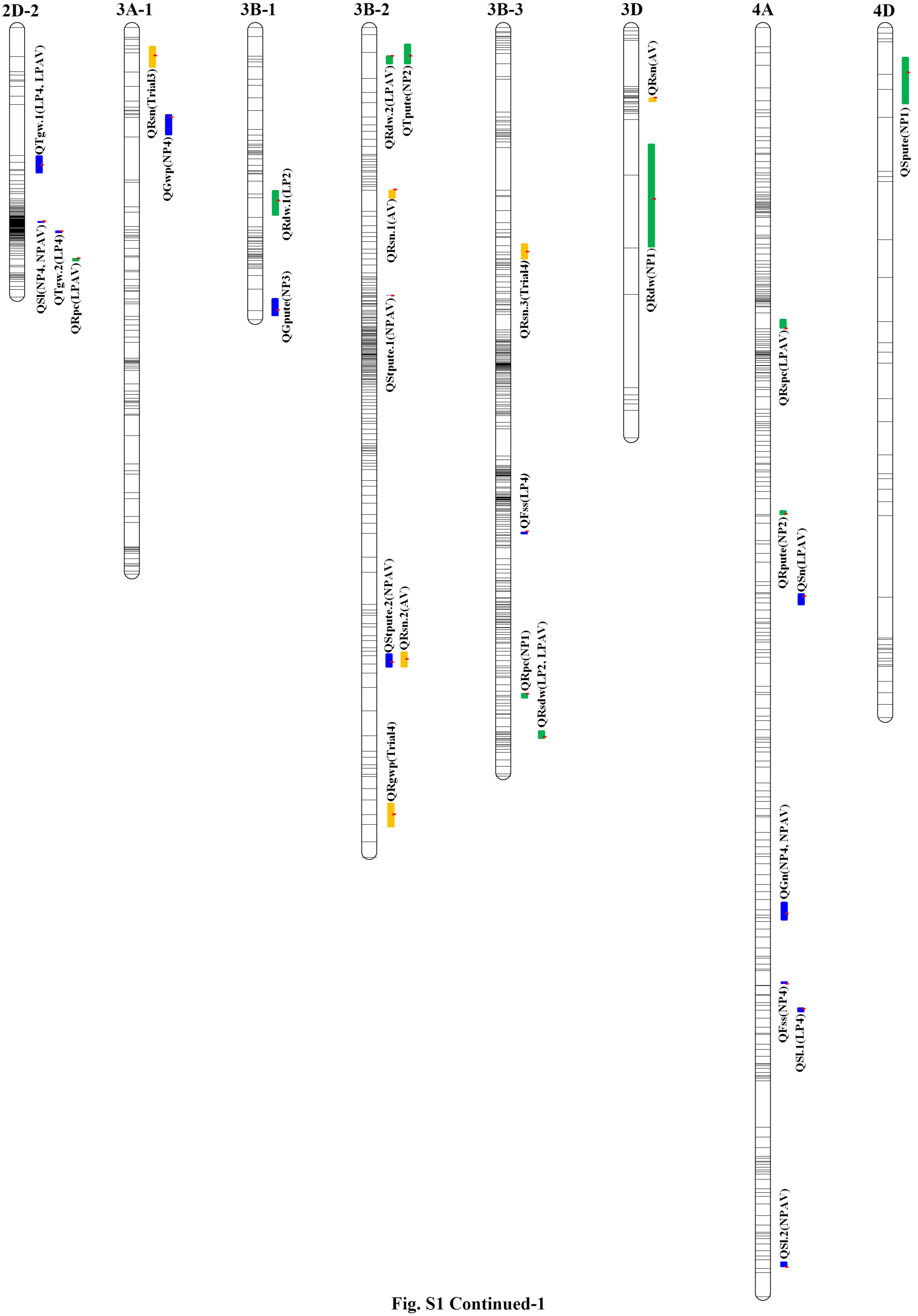

Fig. S1 Continued-1

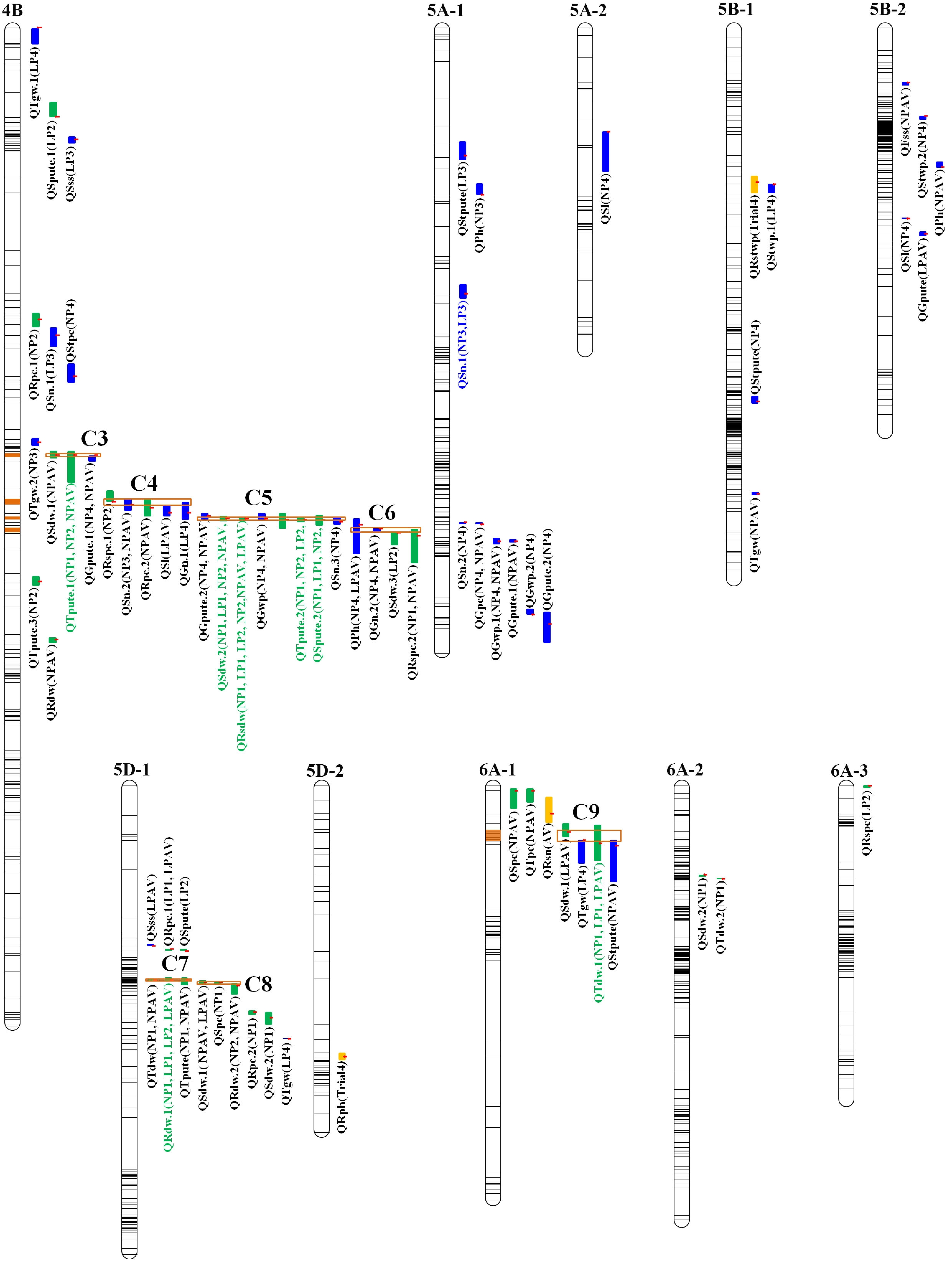

Fig. S1 Continued-2

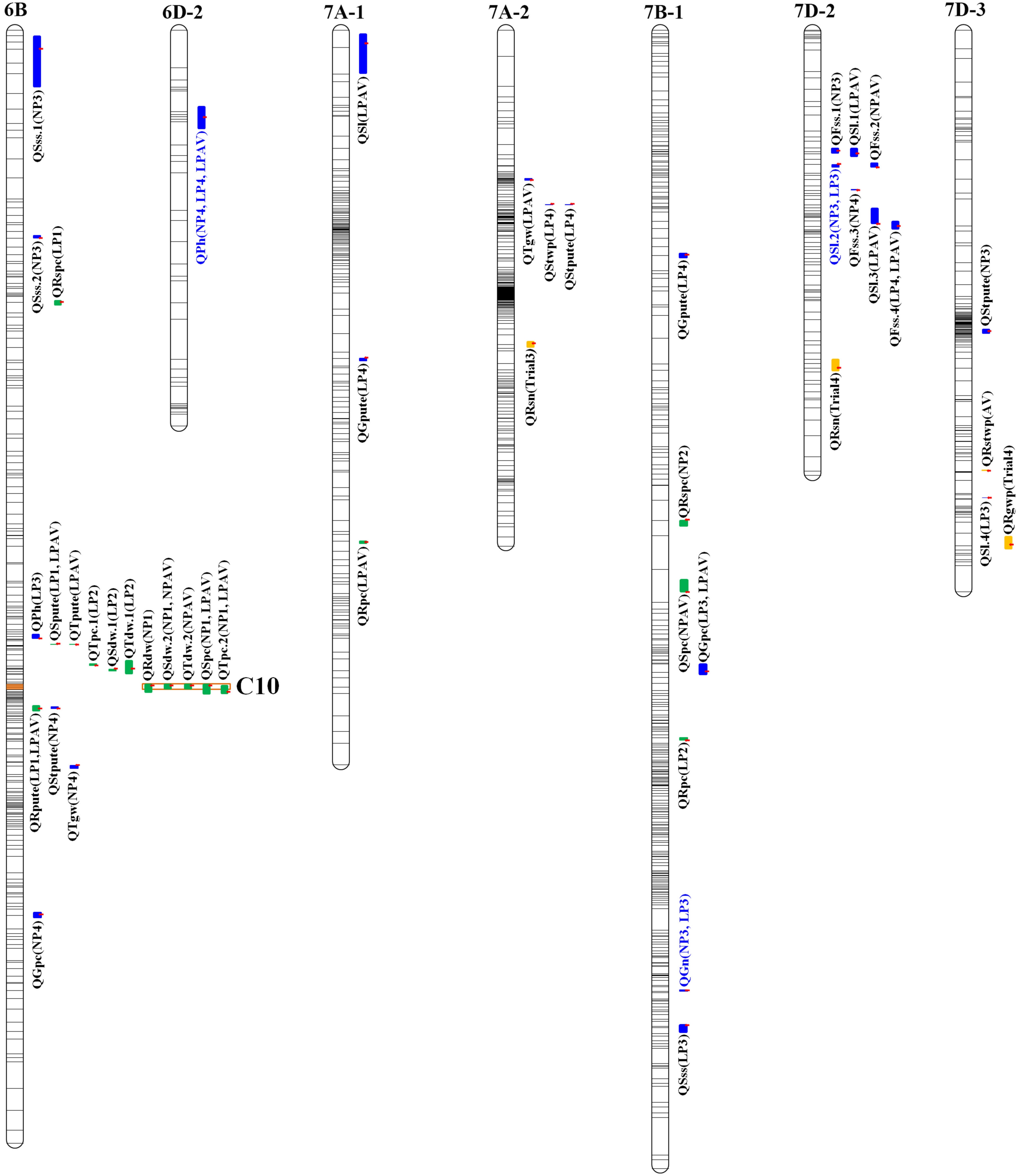

Fig. S1 Continued-3
